# Nuclear restriction of HIV-1 infection by SUN1

**DOI:** 10.1038/s41598-021-98541-4

**Published:** 2021-09-27

**Authors:** Mirjana Persaud, Anastasia Selyutina, Cindy Buffone, Silvana Opp, Daniel A. Donahue, Oliver Schwartz, Felipe Diaz-Griffero

**Affiliations:** 1 Department of Microbiology and Immunology, Albert Einstein College of Medicine, Bronx, NY USA; 2 Department of Virology, Virus & Immunity Unit, Institut Pasteur, Paris, France; 3 Albert Einstein College of Medicine, 1301 Morris Park – Price Center 501, New York, NY 10461 USA

**Keywords:** Virology, Retrovirus, Viral immune evasion, Virus-host interactions

## Abstract

Overexpression of the human Sad-1-Unc-84 homology protein 2 (SUN2) blocks HIV-1 infection in a capsid-dependent manner. In agreement, we showed that overexpression of SUN1 (Sad1 and UNC-84a) also blocks HIV-1 infection in a capsid-dependent manner. SUN2 and the related protein SUN1 are transmembrane proteins located in the inner membrane of the nuclear envelope. The N-terminal domains of SUN1/2 localizes to the nucleoplasm while the C-terminal domains are localized in the nuclear lamina. Because the N-terminal domains of SUN1/2 are located in the nucleoplasm, we hypothesized that SUN1/2 might be interacting with the HIV-1 replication complex in the nucleus leading to HIV-1 inhibition. Our results demonstrated that SUN1/2 interacts with the HIV-1 capsid, and in agreement with our hypothesis, the use of N-terminal deletion mutants showed that SUN1/2 proteins bind to the viral capsid by using its N-terminal domain. SUN1/2 deletion mutants correlated restriction of HIV-1 with capsid binding. Interestingly, the ability of SUN1/2 to restrict HIV-1 also correlated with perinuclear localization of these proteins. In agreement with the notion that SUN proteins interact with the HIV-1 capsid in the nucleus, we found that restriction of HIV-1 by overexpression of SUN proteins do not block the entry of the HIV-1 core into the nucleus. Our results showed that HIV-1 restriction is mediated by the interaction of SUN1/2N-terminal domains with the HIV-1 core in the nuclear compartment.

## Introduction

Early steps of HIV-1 infection involved the delivery of the core into the cytoplasm. The HIV-1 core, which is a supramolecular structure composed of ~ 1800 molecules of capsid, displays a patterned array in its surface that allows the core to interact with multiple cellular factors. The interaction of the core with cellular factors determines the fate of infection. Several proteins that interact with the core are essential for wild type HIV-1 infection: (1) Cyclophilin A promotes HIV-1 infectivity^1–3^, (2) TNPO3 is necessary for infection after nuclear import but before integration^4–10^, (3) Nup153 and RanBP2 are required for nuclear import^11–16^, and (4) CPSF6 is essential for wild type targeting of integration^6,17–20^. The incoming HIV-1 core interacts with all these cellular proteins in order for the virus to achieve productive infection. Overall, the surface of the HIV-1 core is modulating the early steps of HIV-1 infection through the interaction of known and unknown cellular factors. Therefore, finding novel capsid interactors will shed light on the early steps of HIV-1 replication.

Overexpression of the human protein SUN2 was originally discovered to affect HIV-1 infection in a screen that tested the ability of interferon-stimulated genes to block HIV-1 infection^21^. We have previously shown that overexpression of the human SUN2 protein blocks HIV-1 infection in a capsid-dependent manner^22^. These experiments suggested that SUN2 might be interacting with the HIV-1 capsid protein, and that restriction is caused by this interaction.

SUN2 and the related protein SUN1 are transmembrane proteins located in the inner membrane of the nuclear envelope. The nuclear envelope separates the nucleoplasm from the cytoplasm and is composed of inner and an outer membrane. The outer nuclear membrane is contiguous with the endoplasmic reticulum. The two membranes are separated by a thin lumen known as the nuclear lamina or *lamina propia*. The N-terminal domains of SUN1 and SUN2 are located in the nucleoplasm whereas the C-terminal domains are located in the nuclear lamina. The conserved SUN domains, in SUN 1 and SUN2, interact with KASH-domain containing proteins inside the nuclear lamina to form bridges that span both nuclear membranes^23^. These protein bridges have been postulated to be the “Velcro” that links the nucleoskeleton with the cytoskeleton^23^.

We have previously shown that overexpression of the human SUN2 protein blocks HIV-1 infection in a capsid-dependent manner^22^. Here we tested whether overexpression of SUN1 and SUN2 proteins block HIV-1 and other retroviruses. To explore the role of capsid in the ability of SUN1 and SUN2 (SUN1/2) to block HIV-1 infection, we measured the ability of SUN1/2 to bind to the HIV-1 core. Our results demonstrated that SUN1/2 interacts with the HIV-1 capsid in cellular extracts. The use of deletion mutants showed that SUN1/2 proteins bind to the viral capsid using its N-terminal domain, which is located in the nucleoplasm. Furthermore, we found that the N-terminal domain of SUN1/2 governs the ability of the protein to bind HIV-1 capsid and to restrict HIV-1. Interestingly, the ability of SUN1/2 to restrict HIV-1 also correlated with perinuclear localization of these proteins. In addition, we found that restriction of HIV-1 by overexpression of SUN1/2 proteins do not block the entry of the HIV-1 core into the nucleus suggesting that this restriction occurs in the nucleus, which is in agreement with the cellular localization of the N-terminal domain of SUN1/2 proteins. Next, we tested whether cells that do not endogenously expressed SUN1 and SUN2 protein plays a role on HIV-1 infection. To this end, using the CRISPR/Cas9 system, we generated human HAP-1 cells that are knockout for the expression of SUN1, SUN2, or SUN1/SUN2. We found that HAP1 SUN1, SUN2, or SUN1/SUN2 knockout cells were permissive to HIV-1 infection, suggesting that this system is not providing evidence for a functional contribution of SUN1 or SUN2 to HIV-1 infection.

## Results

### Ability of SUN1 to block HIV-1 and other retroviruses

**Figure 1:**
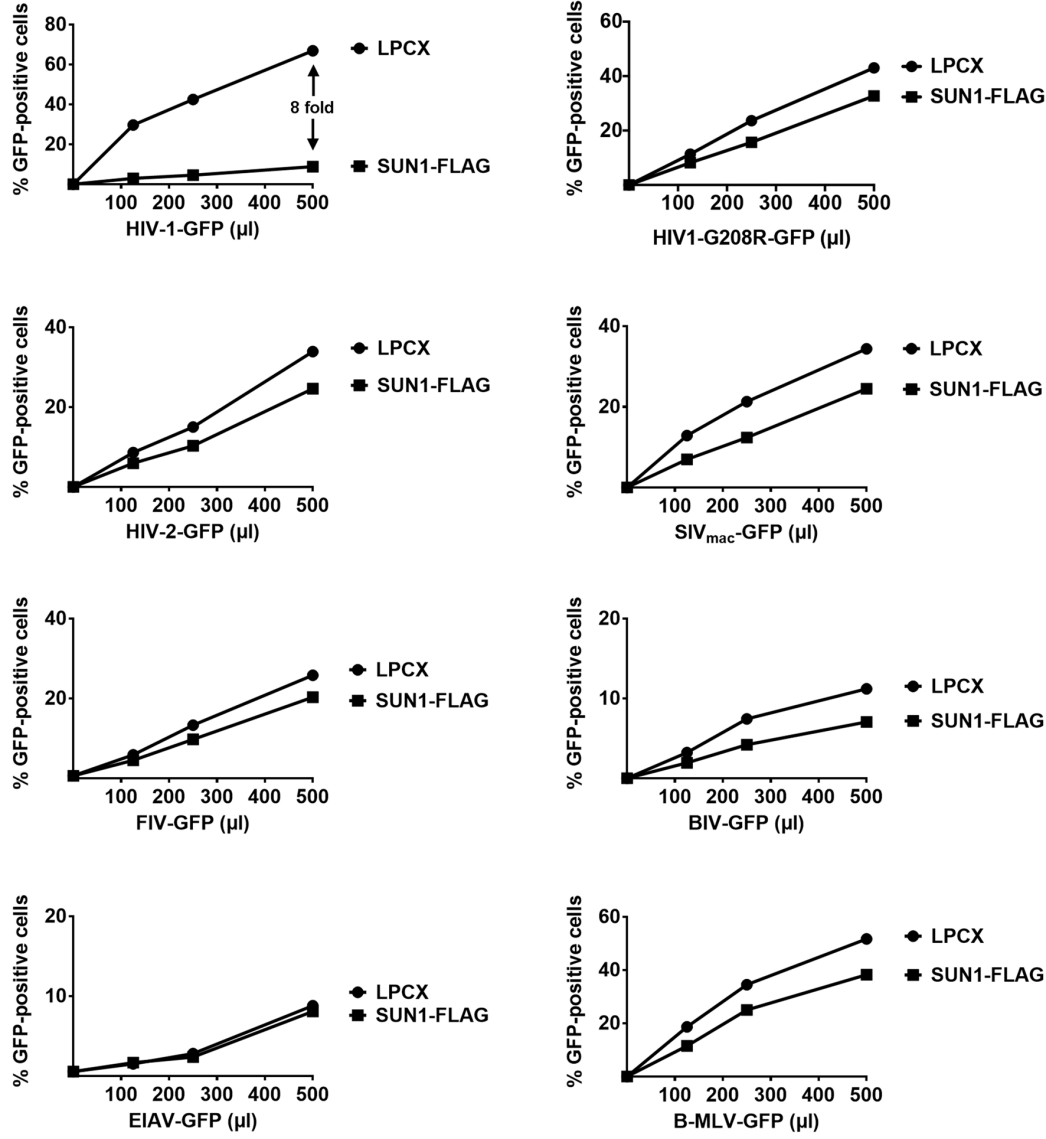
Ability of SUN1 to block HIV-1 and other retroviruses. Human HT1080 fibrosarcoma cells stably expressing wild type SUN1-FLAG or containing the empty vector LPCX were challenged with increasing amounts of HIV-1, HIV-1-G208R, HIV-2, SIV_mac_, FIV, BIV, EIAV, and B-MLV expressing GFP as a reporter. Forty-eight hours post-infection, the percentage of GFP-positive cells was measured using a flow cytometer. Experiments were repeated at least three times and a representative experiment is shown. Fold differences in restriction are shown as the ratio of the area under the curve of the SUN variant to the empty vector pLPCX. Fold values greater than 2 are shown.

To test the ability of SUN1 to block HIV-1 infection, we challenged human HT1080 cells stably expressing SUN1 with increasing amounts of HIV-1-GFP. As shown in Fig. 1, SUN1 potently blocks HIV-1 infection (eightfold). By contrast overexpression of SUN1 was not able to block HIV-1 viruses bearing the capsid mutation G208R. This is in agreement with previous results suggesting that capsid is the viral determinant for the ability of the related protein SUN2 to block HIV-1 infection^22^. Next, we tested whether other retroviruses are restricted by SUN1 overexpression. We found that other primate lentiviruses such as human immunodeficiency virus type 2 (HIV-2), and simian immunodeficiency virus (SIV_mac_), were poorly restricted by SUN1 (Fig. 1). Similarly, feline immunodeficiency virus (FIV), bovine immunodeficiency virus (BIV), equine infectious anemia virus (EIAV), and B-tropic murine leukemia virus (B-MLV), were also poorly restricted by SUN-1 (Fig. 1). Overall, overexpression of SUN1 only restricted HIV-1. Next we decided to study the SUN1 determinants for HIV-1 restriction.

### SUN1 determinants for HIV-1 restriction

**Figure 2:**
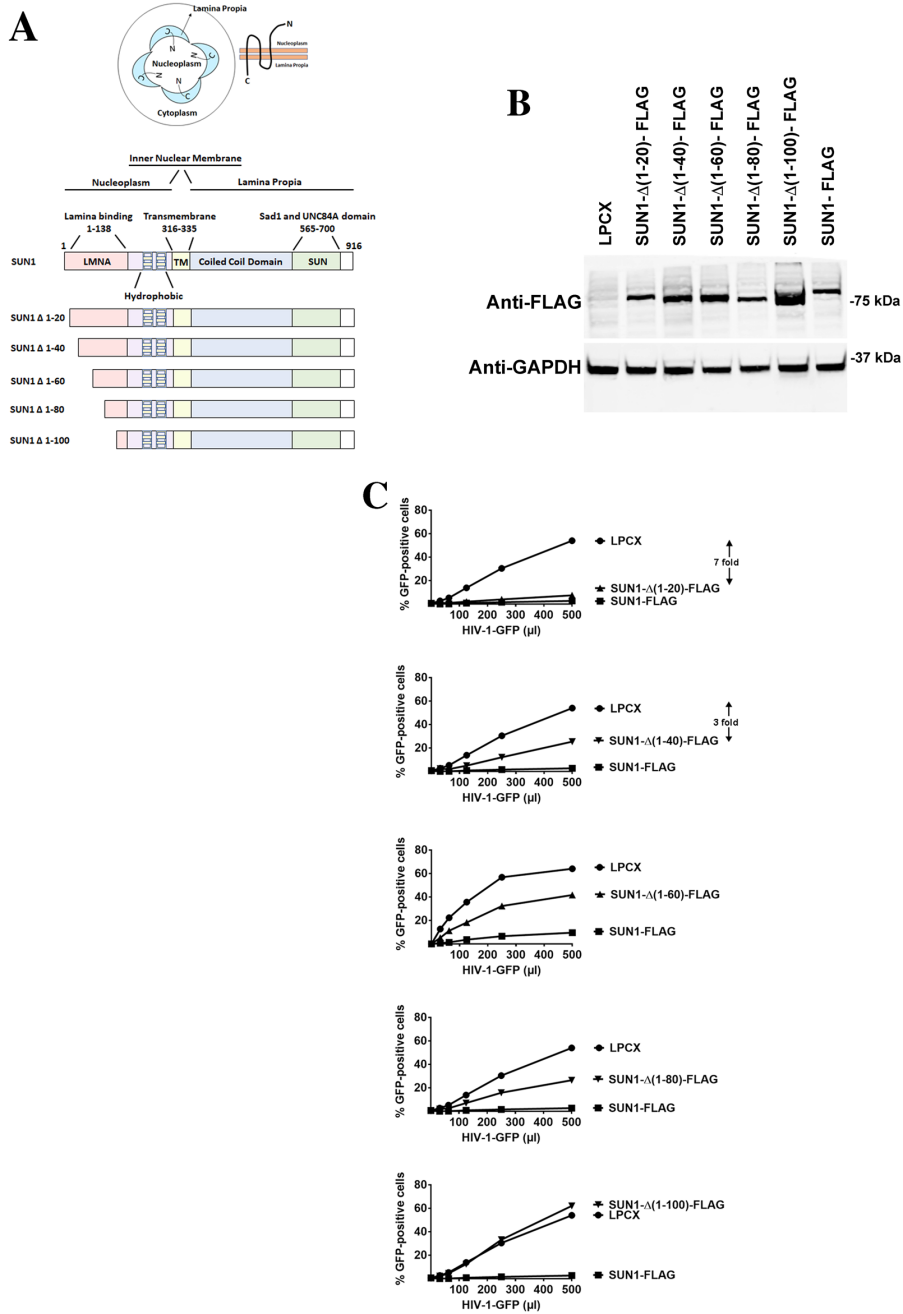
Ability of SUN1 variants to restrict HIV-1 infection. (**A**) SUN1 membrane topology is illustrated in a cell by showing that the N-terminal domain of SUN1 faces the nucleoplasm while the C-terminal domain is in the nuclear lamina or lamina propia. The SUN1 protein is also depicted with the numbers of the amino acid residues at the boundaries of the domains. The SUN1 protein is depicted containing the lamina binding domain (LMNA) (1–138) on the N-terminus, hydrophobic regions, a transmembrane region (316–335), coiled coil domain, and a SUN domain (565–700) on the C-terminus. The different SUN1 N-terminal deletions variants are illustrated. (**B**) Human HT1080 cells stably expressing wild type and mutant SUN1-FLAG proteins. Stable expression of wild type and mutant SUN1 proteins in human HT1080 cells was analyzed by Western blotting using anti-FLAG antibodies. As loading control, extracts were also Western blotted using anti-GAPDH antibodies. (**C**) HIV-1 restriction by wild type and mutant SUN1 proteins. HT1080 cells stably expressing wild type and mutant SUN1 proteins were challenged with increasing amounts of HIV-1-GFP. Forty-eight hours post-infection, the percentage of GFP-positive cells was measured using a flow cytometer. Experiments were repeated at least three times and a representative experiment is shown. Fold differences in restriction are shown as the ratio of the area under the curve of the SUN variant to the empty vector pLPCX. Fold values greater than 2 are shown.

A closer look at the topology of the SUN1 protein suggested that residues 1–315 localized to the nucleoplasm whereas residues 336–916 localized to the nuclear lamina (Fig. 2A). This topology implies that HIV-1 may be interacting with the N-terminal domain of SUN1 in the nucleoplasm (Fig. 2A). Therefore, as shown in Fig. 2A, we created several SUN1 N-terminal deletions. Subsequently, the different SUN1 variants were stably expressed in human HT1080 cells and used to test HIV-1 restriction (Fig. 2B,C). Interestingly, SUN1-∆ (1–20) blocked HIV-1 infection (sevenfold) as potent as the wild type protein (eightfold) suggesting that the first 20 amino acids of the protein are not important for HIV-1 restriction (Fig. 2C and Table 1). Deletions SUN1-∆ (1–40) (threefold), SUN1-∆ (1–60) (< twofold) and SUN1-∆ (1–80) (< twofold) lost potency against HIV-1 (Fig. 2C and Table 1). However, deletion of the first 100 amino acids [SUN1-∆(1–100)] renders the protein inactive against HIV-1 (Fig. 2C and Table 1). Overall these experiments suggested that SUN1 residues 20–100 are important for the ability of SUN1 to block HIV-1 infection. Next we tested whether SUN1 interacts with HIV-1 capsids.

**Table 1:** Phenotypes of SUN1 variants.

SUN1	Restriction of HIV-1^a^	Binding to HIV-1 CA-NC complexes^b^	Localization^c^
WT-FLAG	++	+	PS
∆(1–20)-FLAG	++	+	PS
∆(1–40)-FLAG	+	+	PS + NP
∆(1–60)-FLAG	+	+	PS + NP
∆(1–80)-FLAG	+	+	PS + NP
∆(1–100)-FLAG	−	−	PS + NP

^a^Restriction was measured by infecting cells expressing the indicated SUN1 variant with HIV-1-GFP. After 48 h, the percentage of GFP-positive cells (infected cells) was determined by flow cytometry. “++”: indicates strong restriction, “+” indicates moderate restriction, “−”: indicates absence of restriction.

^b^Binding to the HIV-1 capsid complexes was determined for each SUN1 variant as described in “Methods”. “+”: indicates binding, “−”: indicates no binding.

^c^Wild type and mutant SUN1-FLAG proteins were assayed to determine localization. “PS” indicates Perinuclear staining, “NP” indicates Non-Perinuclear localization. “PS + NP” indicates both perinuclear and non-peri-nuclear staining is observed.

### Capsid binding ability of SUN1

**Figure 3:**
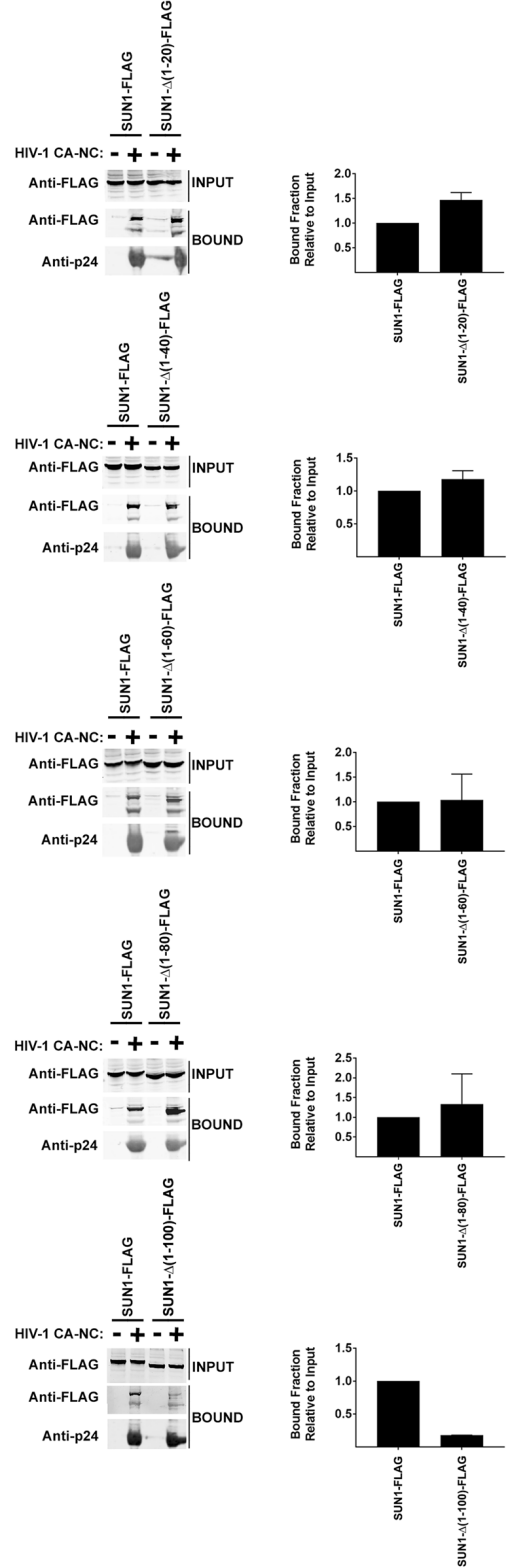
Capsid Binding ability of wild type and mutant SUN1 proteins. HEK293T cells were transiently transfected with plasmids expressing wild type and mutant SUN1-FLAG proteins. Thirty-six hours after transfection, cells were lysed. The lysates were incubated with in vitro assembled HIV-1 CA-NC complexes at room temperature for 1 h. The mixtures were applied onto a 70% sucrose cushion and centrifuged as described in methods. INPUT represents the lysates analyzed by Western blotting before being applied to the 70% cushion. The INPUT fraction was analyzed by Western blotting using anti-FLAG antibodies. The pellet from the 70% cushion (BOUND) was analyzed by Western blotting using anti-FLAG and anti-p24 antibodies. The blots for a representative experiment and the standard deviation for three independent experiments are shown.

Our previous experiments suggested that the N-terminal domain of SUN1 is required for its ability to block HIV-1 infection, which is in agreement with the fact that HIV-1 can only interact with the N-terminus of SUN1 in the nucleoplasm. One hypothesis is that the N-terminal domain of SUN1 is interacting with the HIV-1 replication complex in the nucleus consequentially affecting HIV-1 infection. Because capsid mutations such as G208R abolished the ability of SUN1 to block HIV-1, we tested whether SUN1 binds to the HIV-1 capsid. To this end, we tested whether SUN1 binds to in vitro assembled HIV-1 CA-NC complexes, which recapitulates the surface of the HIV-1 core^24^. As shown in Fig. 3 and Table 1, SUN1 binds to in vitro assembled HIV-1 CA-NC complexes. Interestingly, deletions SUN1-∆(1–40), SUN1-∆(1–60) and SUN1-∆(1–80) bind to in vitro assembled HIV-1 CA-NC complexes similar to wild type SUN1(Fig. 3 and Table 1). By contrast, deletion SUN1-∆(1–100) lost its ability to bind in vitro assembled HIV-1 CA-NC complexes (Fig. 3 and Table 1). These experiments showed that loss of binding correlates with loss of restriction. This is in agreement with our hypothesis that the N-terminal domain of SUN1 is important for its ability to bind capsid and restrict HIV-1 infection.

### Ability of SUN2 to block HIV-1 and other retroviruses

**Figure 4:**
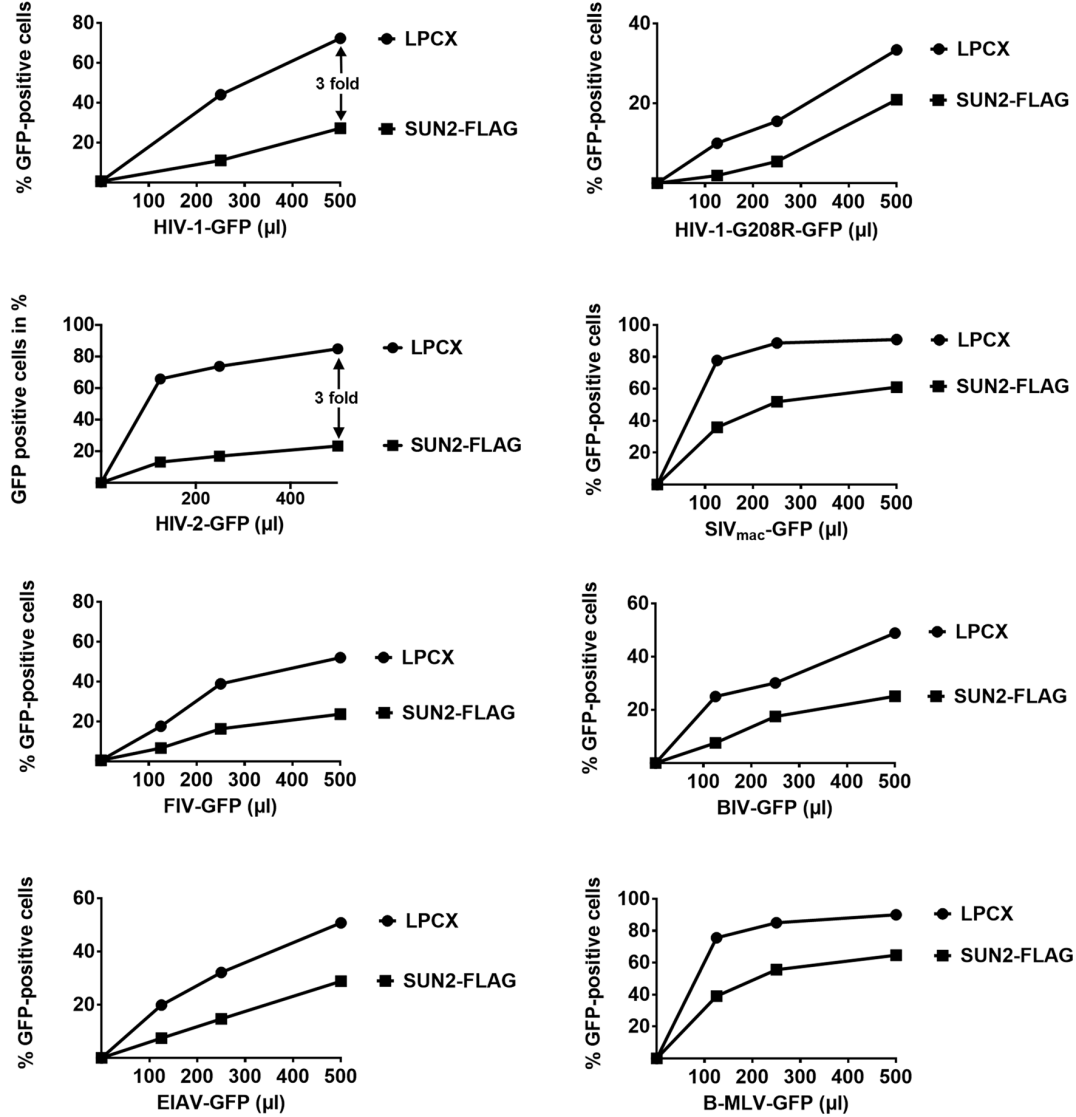
Ability of SUN2 to block HIV-1 and other retroviruses. Human HT1080 fibrosarcoma cells stably expressing wild type SUN2-FLAG or containing the empty vector LPCX were challenged with increasing amounts of HIV-1, HIV-1-G208R, HIV-2, SIV_mac_, FIV, BIV, EIAV, and B-MLV expressing GFP as a reporter. Forty-eight hours post-infection, the percentage of GFP-positive cells was measured using a flow cytometer. Experiments were repeated at least three times and a representative experiment is shown. Fold differences in restriction are shown as the ratio of the area under the curve of the SUN variant to the empty vector pLPCX. Fold values greater than 2 are shown.

Next we tested the ability of SUN2 to block HIV-1 infection. As we have previously shown^22^, SUN2 blocks HIV-1 infection (threefold). Although overexpression of SUN2 blocks HIV-1 infection (Fig. 4), we observed that this block is less potent when compared to SUN1. Like SUN1, SUN2 poorly affected the infection of HIV-1 bearing the capsid mutation G208R, which is in agreement with our previous results suggesting that capsid mutations overcome the HIV-1 restriction imposed by overexpression of SUN2^22^. Like in the case of SUN1, these experiments suggested that capsid is the viral determinant for the restriction of SUN2. Next we tested whether other retroviruses are restricted by SUN2 overexpression. We found that SUN2 modestly restricts HIV-1 and HIV-2 (threefold), but not SIV_mac_ (Fig. 4). Overexpression of SUN2 poorly affected infection by FIV, BIV, EIAV, and B-MLV viruses.

### SUN2 determinants for HIV-1 restriction

**Figure 5:**
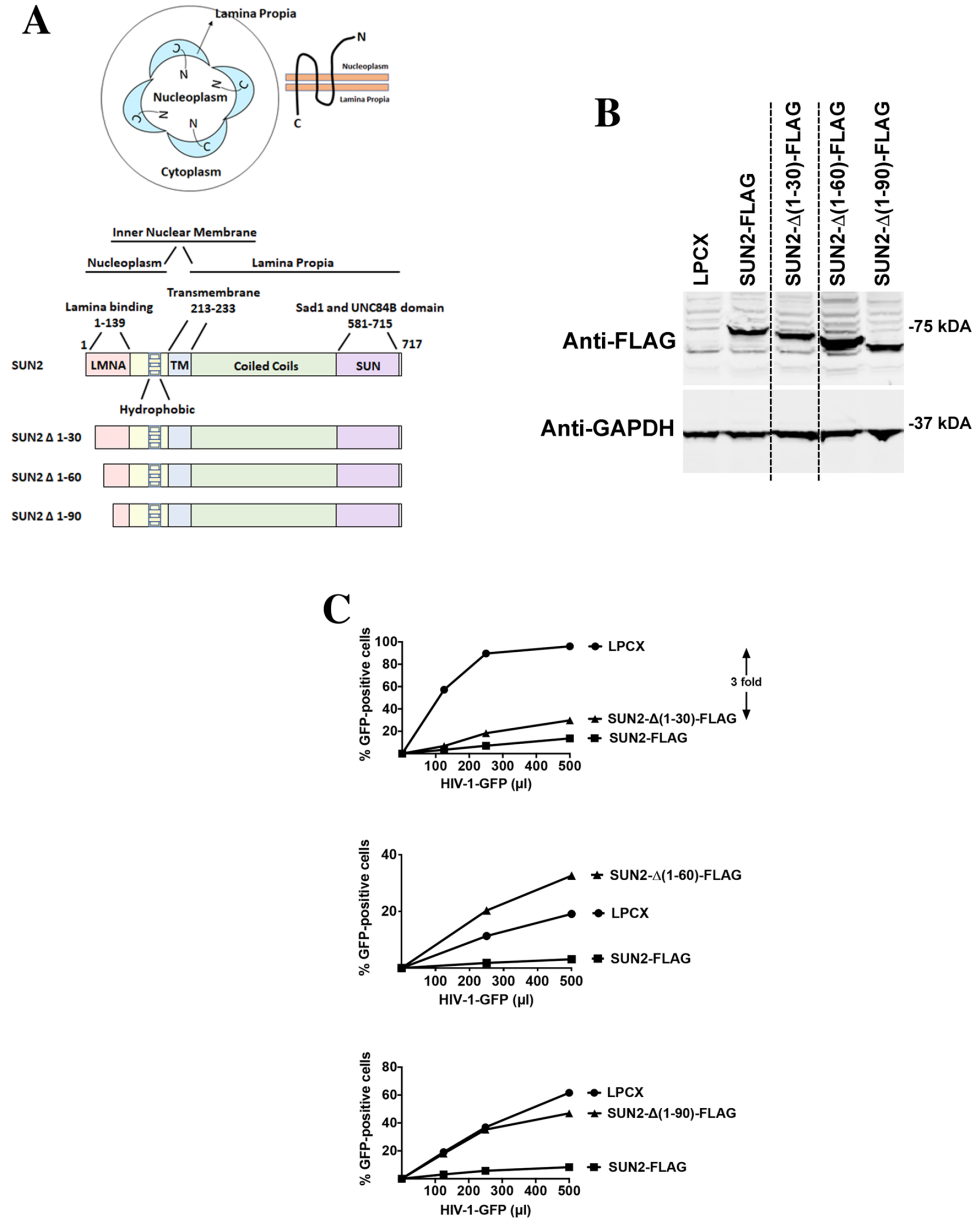
Ability of SUN2 variants to restrict HIV-1 infection. (**A**) SUN2 membrane topology is illustrated in a cell by showing that the N-terminal domain of SUN2 faces the nucleoplasm while the C-terminal domain is in the nuclear lamina or lamina propia. The SUN2 protein is also depicted with the numbers of the amino acid residues at the boundaries of the domains. The SUN2 protein is depicted containing the lamina binding domain (LMNA) (1–139) on the N-terminus, hydrophobic regions, a transmembrane region (213–233), coiled coil domain, and a SUN domain (581–715) on the C-terminus. The different SUN2 N-terminal deletions variants are illustrated. (**B**) Human HT1080 cells stably expressing wild type and mutant SUN2 proteins. Stable expression of wild type and mutant SUN2 proteins in human HT1080 cells was analyzed by Western blotting using anti-FLAG antibodies. As loading control, extracts were also Western blotted using anti-GAPDH antibodies. (**C**) HIV-1 restriction by wild type and mutant SUN2 proteins. HT1080 cells stably expressing wild type and mutant SUN2 proteins were challenged with increasing amounts of HIV-1-GFP. Forty-eight hours post-infection, the percentage of GFP-positive cells was measured using a flow cytometer. Experiments were repeated at least three times and a representative experiment is shown. Fold differences in restriction are shown as the ratio of the area under the curve of the SUN variant to the empty vector pLPCX. Fold values greater than 2 are shown.

Similar to SUN1, the topology of SUN2 implies that HIV-1 may be interacting with the N-terminal domain of SUN2 in the nucleoplasm (Fig. 5A). Therefore, as shown in Fig. 5A, we created several SUN2 N-terminal deletions. Subsequently, the different SUN2 variants were stably expressed in human HT1080 cells and used to test HIV-1 restriction (Fig. 5B,C). Interestingly, SUN2-∆(1–30) blocked HIV-1 infection (threefold) as potent as the wild type protein (threefold) suggesting that the first 30 amino acids of the protein are not important for HIV-1 restriction(Fig. 5C and Table 2). Deletions SUN2-∆(1–60) and SUN2-∆(1–90) render the protein inactive against HIV-1 (Fig. 5C and Table 2). Overall these experiments suggested that SUN2 residues 30–60 are required for its ability to block HIV-1 infection. On the next section, we tested whether SUN2 interacts with HIV-1 capsids.

**Table 2:** Phenotypes of SUN2 variant.

SUN2	Restriction of HIV-1^a^	Binding to HIV-1 CA-NC complexes^b^	Localization^c^
WT-FLAG	++	++	PS
∆(1–30)-FLAG	++	+	PS + NP
∆(1–60)-FLAG	−	+/−	PS + NP
∆(1–90)-FLAG	−	−	PS + NP

^a^Restriction was measured by infecting cells expressing the indicated SUN2 variant with HIV-1-GFP. After 48 h, the percentage of GFP-positive cells (infected cells) was determined by flow cytometry. “++”: indicates strong restriction, “+” indicates moderate restriction, “−”: indicates absence of restriction.

^b^Binding to the HIV-1 capsid complexes was determined for each SUN2 variant as described in “Methods”. “+”: indicates binding, “−”: indicates no binding, “+/−” decreased binding.

^c^Wild type and mutant SUN2-FLAG proteins were assayed to determine localization. “PS” indicates Perinuclear staining, “NP” indicates Non-Perinuclear localization. “PS + NP” indicates both perinuclear and non-peri-nuclear staining is observed.

### Capsid binding ability of SUN2

**Figure 6:**
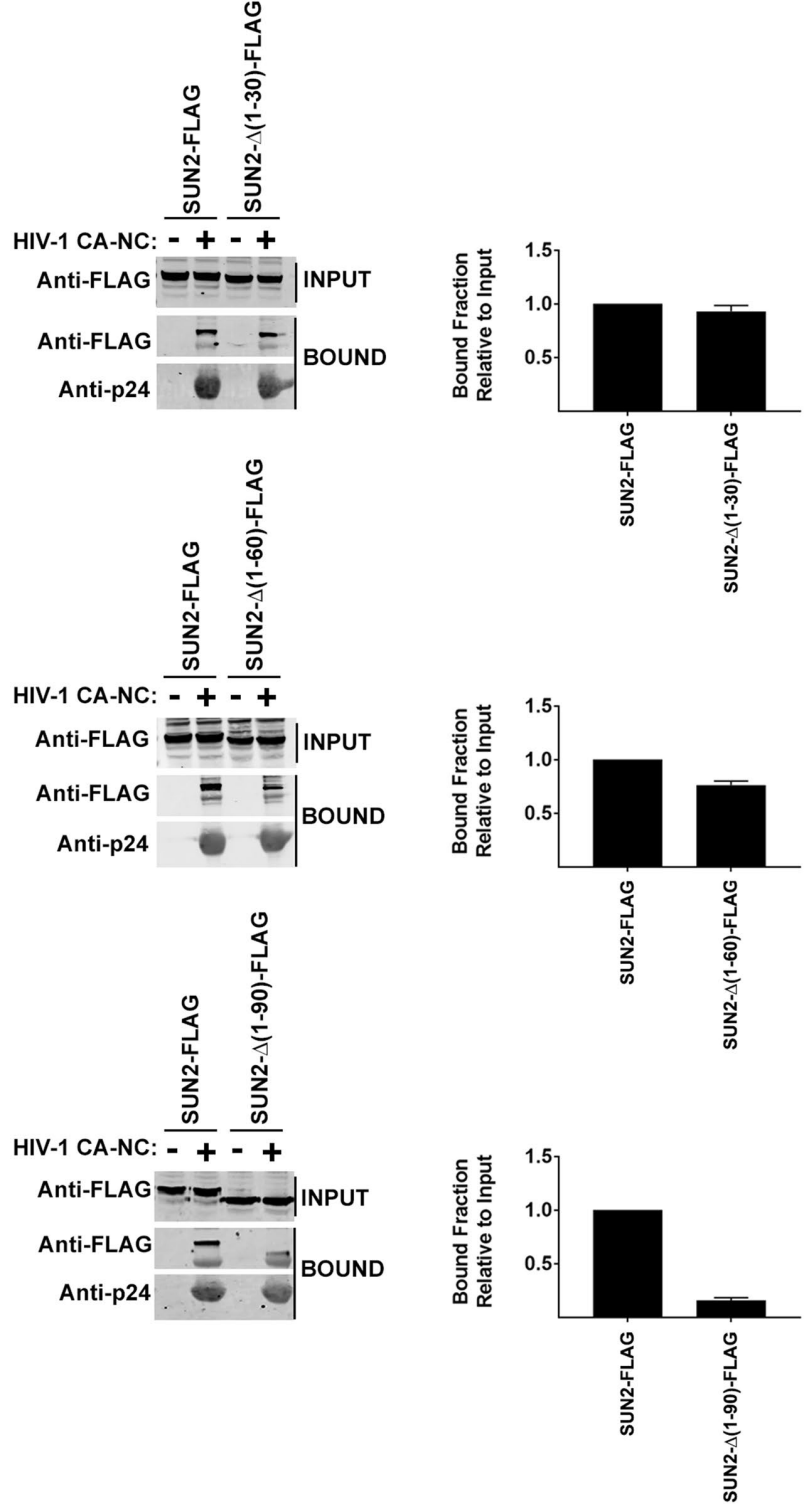
Capsid Binding ability of wild type and mutant SUN2 proteins. HEK293T cells were transiently transfected with plasmids expressing wild type and mutant SUN2-FLAG proteins. Thirty-six hours after transfection, cells were lysed. The lysates were incubated with in vitro assembled HIV-1 CA-NC complexes at room temperature for 1 h. The mixtures were applied onto a 70% sucrose cushion and centrifuged as described in methods. INPUT represents the lysates analyzed by Western blotting before being applied to the 70% cushion. The INPUT fraction was analyzed by Western blotting using anti-FLAG antibodies. The pellet from the 70% cushion (BOUND) was analyzed by Western blotting using anti-FLAG and anti-p24 antibodies. The blots for a representative experiment and the standard deviation for three independent experiments are shown.

Our previous experiments suggested that the N-terminal domain of SUN2 is required for its ability to block HIV-1 infection, which is in agreement with the fact that the N-terminus of SUN2 is localized to the nucleoplasm. In the same manner as SUN1, we tested the hypothesis that the N-terminal domain of SUN2 is interacting with the HIV-1 replication complex in the nucleus consequentially affecting HIV-1 infection. Because capsid mutations such as G208R and P207S abolished the ability of SUN2 to block HIV-1^22^, we also tested whether SUN2 binds to the HIV-1 capsid. As shown in Fig. 6, SUN2 binds to in vitro assembled HIV-1 CA-NC complexes. Deletions SUN2-∆(1–30) retained binding to in vitro assembled HIV-1 CA-NC complexes. However, deletions SUN1-∆(1–60) and SUN2-∆(1–90) decreased their ability to bind in vitro assembled HIV-1 CA-NC complexes. In the case of the deletion SUN1-∆(1–60), we observed residual binding when compared to SUN2-∆(1–90). These experiments are in agreement with our hypothesis that the N-terminal domain is important for the ability of SUN2 to bind capsid and restrict HIV-1 infection.

### Capsid Binding ability of SUN1 and SUN2 to mutants G208R and P207S

**Figure 7:**
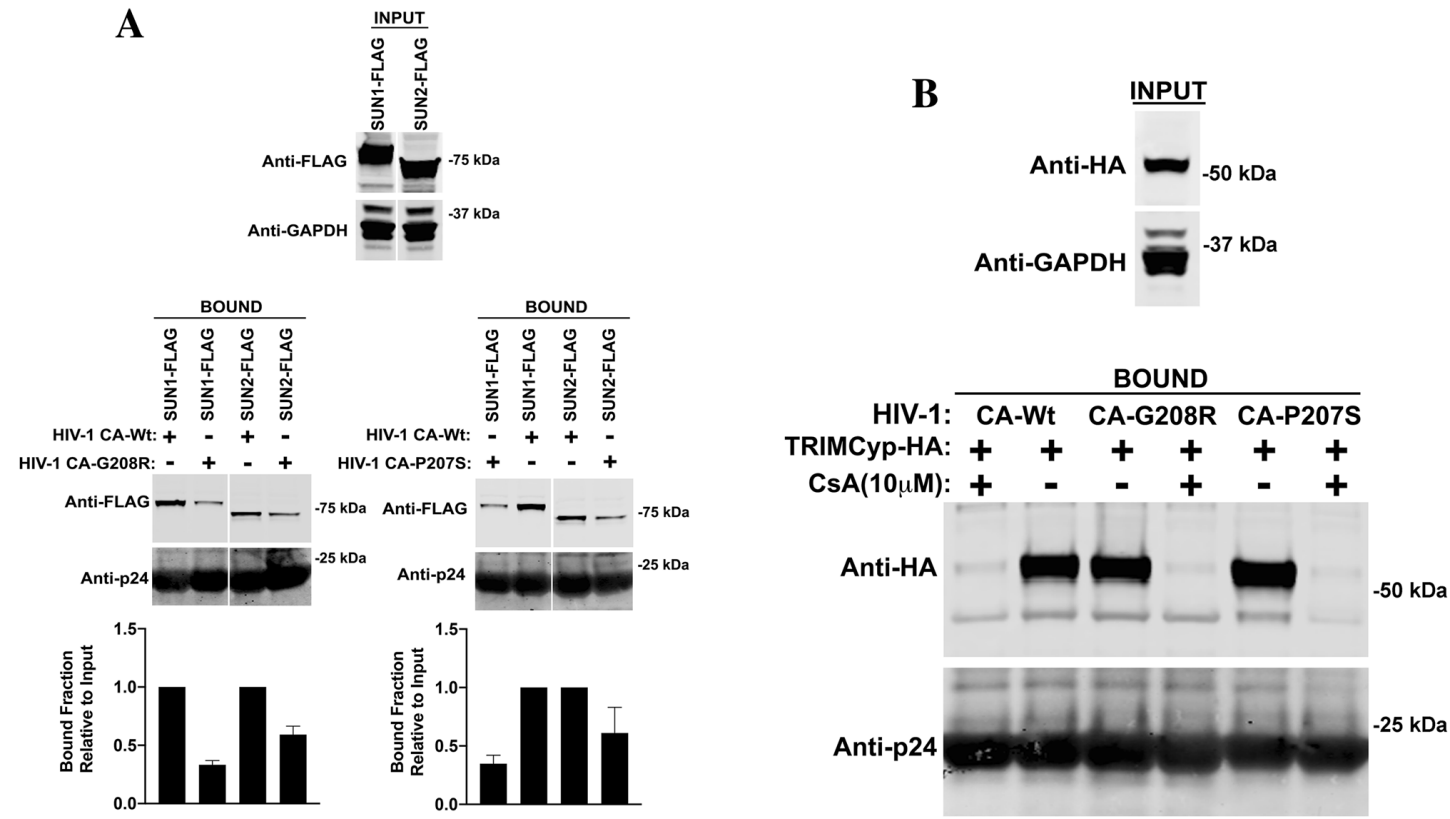
Binding of SUN1 and SUN2 to G208R and P207S capsid mutants. (**A**) HEK293T cells were transiently transfected with plasmids expressing wild type SUN1-FLAG and SUN2-FLAG. Twenty-four hours after transfection, cells were lysed. The lysates were incubated with in vitro assembled HIV-1-CA WT, HIV-1-CA G208R, and HIV-1-CA P207S complexes at room temperature for 1 h. The mixture was centrifuged to pellet the capsid as described in “Methods”. (**B**) HEK293T cells were transiently transfected with TRIMCyp-HA as a control for binding. Twenty-four hours after transfection, cells were lysed. Lysates were incubated in the presence of 10 μM cyclosporine A or DMSO vehicle control. In vitro assembled HIV-1-CA WT, HIV-1-CA G208R, and HIV-1-CA P207S complexes were added to the pre-treated lysates for 1 h at room temperature. INPUT represents the lysates analyzed by Western blotting prior to the addition of the capsid. The INPUT fraction was analyzed by Western blotting using anti-FLAG or anti-HA antibodies respectively. The pellet (BOUND) fraction was analyzed by Western blotting using anti-FLAG and anti-p24 antibodies. The blots for a representative experiment and the standard deviations for three independent experiments are shown.

Next we tested the ability of SUN1 and SUN2 to bind capsid mutants G208R and P207S. In agreement with the inability of SUN1 and SUN2 to block infection of HIV-1 viruses bearing the capsid changes G208R or P207S, we saw that SUN1 and SUN2 showed decreased binding to capsid bearing G208R or P207S changes when compared to wild type (Fig. 7A). These experiments demonstrated that the inability of SUN1 and SUN2 to block HIV-1 viruses bearing capsid changes G208R and P207S is due to the lose of capsid binding. To control for the *bona fide* folding of the capsid mutants, we tested the ability of capsid mutants to interact with TRIMCyp in presence or absence of cyclosporine A (CsA). As shown in Fig. 7B, TRIMCyp was able to interact with capsid mutants at wild type levels. Overall these results correlate SUN1/2 binding with restriction.

### Subcellular localization of SUN1 and SUN2 variants

**Figure 8:**
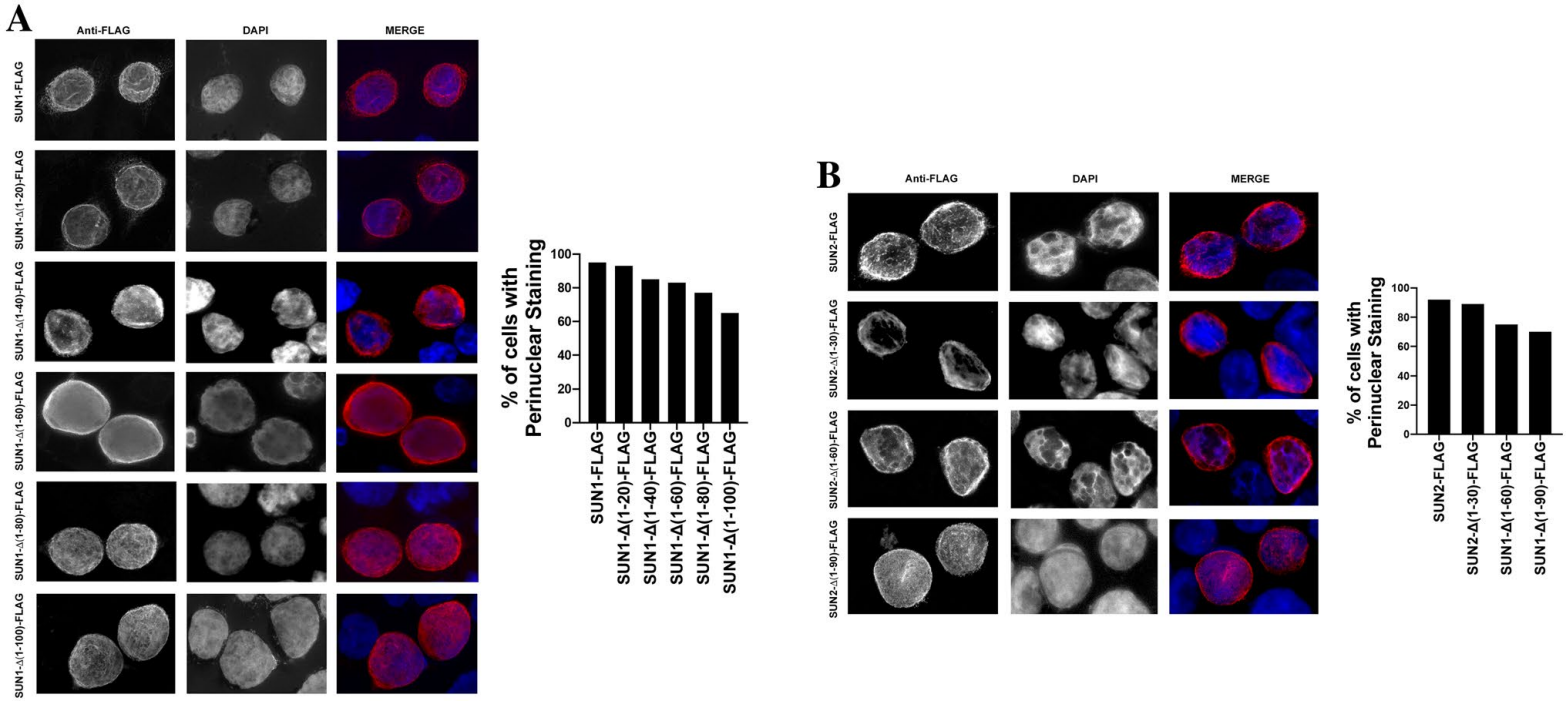
Subcellular localization of SUN1 and SUN2 variants. The subcellular localization of SUN1-FLAG (**A**) and SUN2-FLAG (**B**) was studied in HeLa cells. Human HeLa cells transiently transfected with the indicated constructs were fixed and permeabilized. Subsequently, SUN1/2 proteins were stained using anti-FLAG antibodies conjugated to Alexa Fluor 594. The nuclei was stained by using DAPI (blue). Perinuclear staining was quantified by examining 50 cells for SUN1-FLAG, SUN2-FLAG and mutants in three independent experiments; results are shown as percentage of cells showing perinuclear staining.

Previous experiments have defined that SUN1 and SUN2 localized to the lamina *propia*^25^. To understand weather the subcellular localization of SUN1 and SUN2 are important for their ability to block HIV-1 infection, we analyzed subcellular localization of the different SUN1 and SUN2 variants. As shown in Fig. 8, SUN1 and SUN2 showed distinct perinuclear localization, which according to the literature, corresponds to the lamina *propia*. Interestingly, the localization of the deletion mutant SUN1-∆(1–20) resembles the subcellular localization of the wild type SUN1 protein (Fig. 8A), suggesting that this deletion did not lose wild type localization. Although all other SUN1 deletions showed a perinuclear localization, they also displayed a more intense nucleoplasm staining (Fig. 8A). SUN2 deletion constructs showed a perinuclear staining similar to wild type SUN2. In addition, deletion constructs showed a more intense nucleoplasm staining (Fig. 8B). Interestingly, all study deletions localized to the perinuclear region suggesting that these constructs were not majorly affected by the deletions, indirectly indicating that folding was not greatly disrupted. Perinuclear staining was quantified by examining 50 cells for SUN1-FLAG, SUN2-FLAG and mutants (Fig. 8A,B). Our results showed that for each sequential deletion mutant, there is some nucleoplasmic staining in addition to the pre-dominant peri-nuclear staining.

### Inhibition of HIV-1 infection by SUN1 does prevent the entry of capsid into the nucleus

**Figure 9:**
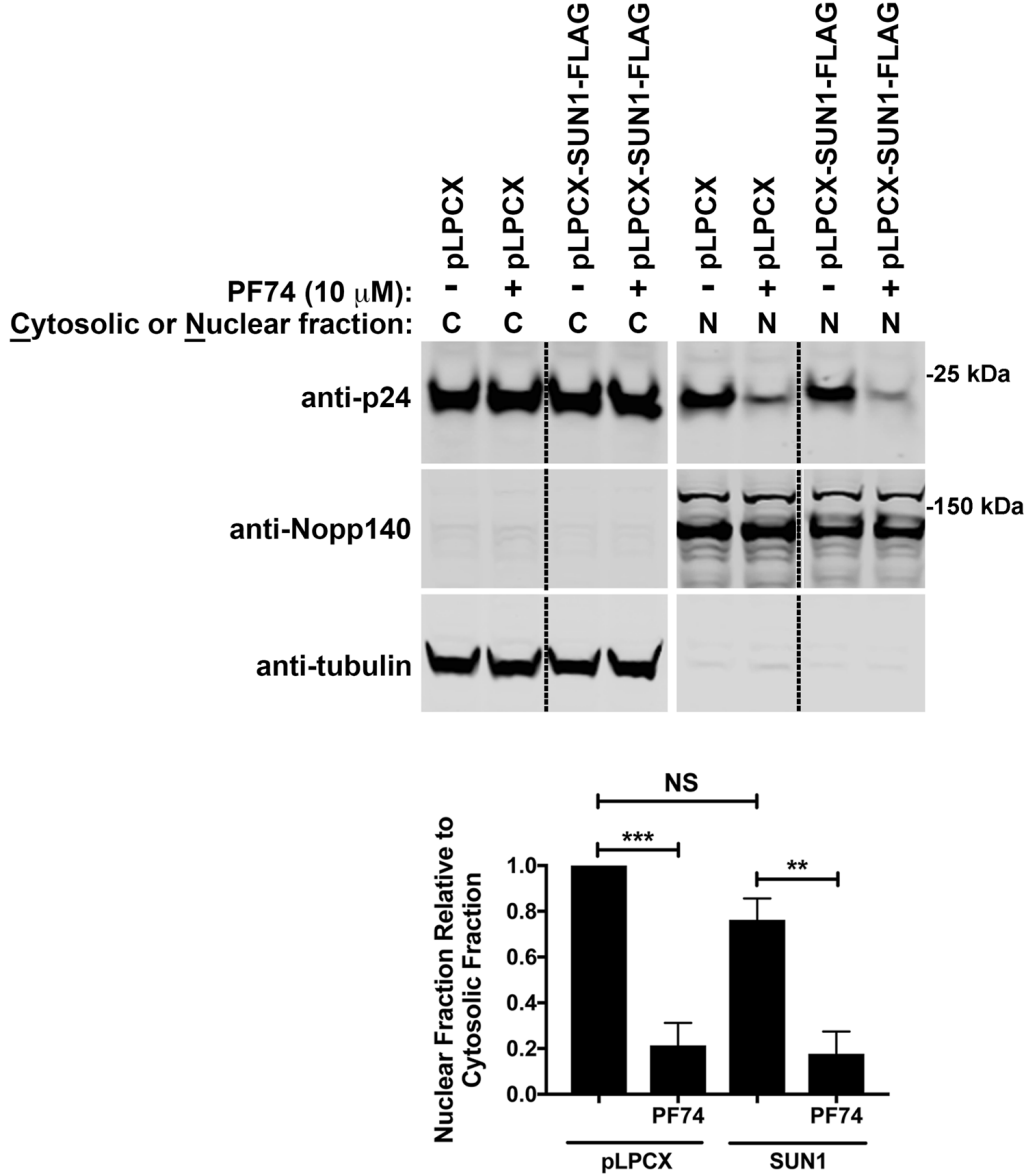
Nuclear Import of capsid in SUN1 overexpressed cells. Human HT1080 cells, stably expressing SUN1-FLAG or containing the LPCX empty vector control, were infected with wild-type HIV-1-GFP at an MOI of 2 in the presence of 10uM PF74, or DMSO vehicle control for 8 h. Cells were separated into nuclear and cytosolic fractions and analyze for capsid content by western blotting using anti-p24, anti-Nopp140 and anti-α-Tubulin antibodies. The ratio of nuclear to cytosolic capsid for three independent experiments with standard deviations is shown. **p < 0.001; ***p < 0.0005; NS, not significant (as determined by unpaired t test).

Overexpression of SUN1 in human cells potently inhibit HIV-1 infection after reverse transcription but prior to integration^22^. Because the occurrence of reverse transcription correlates with entry of capsid into the nucleus^26–28^, and the SUN1 domain that interacts with capsid is in the nucleoplasm; we tested whether SUN1 inhibition of HIV-1 allows the entry of capsid into the nucleus. For this purpose, using our published methodology^26^, we tested whether capsid is imported into the nucleus of HIV-1 restricted cells by SUN1. To this end, human HT1080 cells expressing SUN1 were challenged with HIV-1 using an MOI = 2. Eight hours post infection, cells were separated into cytosolic (C) and nuclear (N) fractions. C and N fractions were analyzed for capsid content using anti-P24 antibodies (Fig. 9). To ensure *bona fide* origin of the cellular fractions, we performed Western blot analysis using anti-Nopp140 and anti-tubulin as nuclear and cytosolic markers, respectively. As shown on Fig. 9, we found that expression of SUN1 does not inhibit the entry of capsid into the nucleus. As controls, we used the small molecule PF74 that potently blocks the entry of capsid into the nucleus^26^. These experiments suggest that HIV-1 restriction by SUN1 occurs in the nucleus, which is in agreement with findings showing that the region of SUN1 that interact with capsid is in the nucleoplasm. Overall these experiments suggest that the restriction of HIV-1 by overexpression of SUN1 occurs in the nuclear compartment.

### Endogenous expression of SUN1/SUN2 is not required for wild type HIV-1 infection in human haploid HAP-1 cells

**Figure 10:**
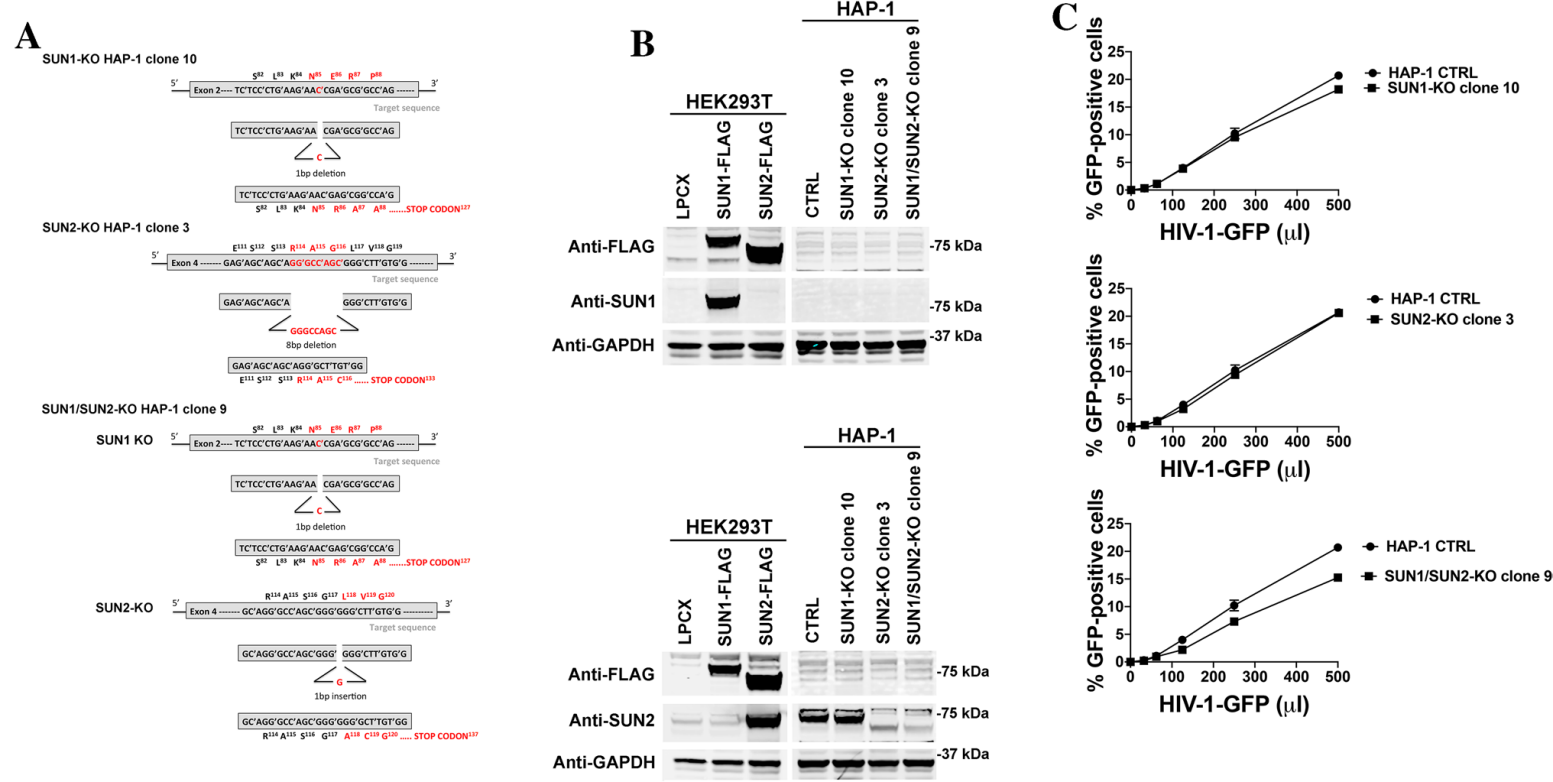
Endogenous SUN2 is not required for wild type HIV-1 infection in human HAP-1 cells. (**A**) Construction of human HAP-1 cells that do not express SUN1, SUN2, or both (SUN1/SUN2). HAP-1 cells were transiently transfected with CRISPR-Cas9 system using a specific RNA guide that targets the exon 2 of SUN1 and/or exon 4 of SUN2. Sequencing of SUN1, SUN2, and SUN1/2 KO clones revealed frame shifts that resulted in the creation of early stop codons. (**B**) HAP-1 KO cells were tested for expression of endogenous SUN1 and SUN2 by Western blotting using anti-SUN1 and SUN2 antibodies. To test for the *bona fide* recognition of SUN1 and SUN2 by anti-SUN1/2 antibodies, we performed similar Western blots using lysates from human HEK293T cells that were transiently transfected with SUN1-FLAG or SUN2-FLAG proteins. As loading control, extracts were also Western blotted using anti-GAPDH antibodies. (**C**) HIV-1 infection of HAP-1 SUN1 KO (clone 10), SUN2 KO (clone 3), and SUN1/SUN2 KO (clone 9) cell lines. HAP-1 control cells and HAP-1 KO cell lines were challenged with increasing amounts of HIV-1-GFP. Forty-eight hours post-infection, the percentage of GFP-positive cells was measured using a flow cytometer. Experiments were repeated at three times and a representative experiment is shown. Fold differences in restriction are shown as the ratio of the area under the curve of the SUN variant to the empty vector pLPCX. Fold values greater than 2 are shown.

Although our work shows that overexpression of SUN1 and SUN2 block HIV-1 infection, we did not yet address whether endogenous expression of these proteins are important for HIV-1 infection. To directly address this question, we knockout the expression of SUN1 and/or SUN2 in human haploid HAP-1 cells by using the CRISPR/Cas9 system. As shown in Fig. 10A, sequencing of the SUN1 and/or SUN2 alleles revealed deletions that resulted in the generation of premature stop codons (Fig. 10A). Furthermore, we tested expression of endogenous SUN1 and SUN2 in the KO HAP-1 cells. As shown in Fig. 10B, HAP-1 cell lines containing the premature stop codons did not expressed SUN1/2 proteins. Next we tested whether HAP-1 cells that do not express SUN1 and/or SUN2 were infected by HIV-1-GFP (Fig. 10B). As a control, we used a clone that showed an intact sequence for SUN1 and SUN2 (HAP-1 CTRL). Interestingly, knocking out the expression of these genes did not affect HIV-1 infection (Fig. 10B,C). These experiments suggested that expression of SUN1 and/or SUN2 is not important for HIV-1 infection of HAP-1 cells.

## Discussion

This work and others have established that overexpression of SUN1 and SUN2 blocks HIV-1 infection, and this process depends upon an intact capsid protein^21,22,29^. The present work showed that SUN1/2 is interacting with capsid in the nuclear compartment to achieve HIV-1 restriction. To this end, we tested the ability of SUN1 and SUN2 to bind to in vitro assembled HIV-1 CA-NC complexes. Our data showed that SUN1/2 proteins in cell lysates interact with the HIV-1 core, in agreement with previous findings^29^. This interaction is likely to occur in the nucleoplasm where the N-terminal of SUN1/2 is located. One possibility is that the interaction between the N-terminal domain of SUN1 or SUN2 with the pre-integration complex negatively affects infection by changing the localization of the core in the nuclear compartment.

Since the N-terminal of SUN1 or SUN2 is the only segment of the protein that might be interacting with HIV-1, we performed SUN1/2N-terminal deletions and tested for restriction. In agreement with the hypothesis that the N-terminal domain of SUN1/2 are important for restriction, we found that deletion of N-terminal residues renders SUN1/2 inactive against HIV-1 infection. Our mapping experiments revealed that residues 20–100 and 30–60 are important for HIV-1 restriction of SUN1 and SUN2, respectively. These experiments demonstrated that the N-terminal region of SUN1/SUN2 is important for the ability of the protein to block HIV-1. Similar results for SUN1 were observed by others^29,30^.

Next, we investigated whether a correlation between restriction and capsid binding exists. Interestingly, N-terminal deletion mutants that did not bind capsid did not restrict HIV-1. In addition, we demonstrated that capsid mutations G208R and P207S prevent the ability of capsid to bind to SUN1/2 proteins; this is in agreement with the inability of SUN1/2 to restrict HIV-1 viruses bearing changes G208R and P207S. These results correlated binding to capsid with HIV-1 restriction. Overall this is in agreement with the hypothesis that the N-terminal domain of SUN1/2 is interacting with capsid during infection and that this interaction is important for restriction.

SUN1/2 proteins mainly localize to the *lamina* propia. To understand whether the localization of SUN1/2 correlates with restriction, we investigated the subcellular localization of SUN1/2N-terminal deletion mutants and correlated this to restriction. Our observations revealed that intact restriction is only achieved by an intact localization of SUN1/2. Although N-terminal deletion mutants remain in the lamina propia (perinuclear localization), we observed that HIV-1 restriction by SUN1 and SUN2 correlates with perinuclear localization. Deletion mutants that exhibit perinuclear and nonperinuclear localization were less or none restrictive to HIV-1. Therefore, SUN1/2 restriction correlates with capsid binding and perinuclear localization. Similar localization for SUN1 deletion mutants was observed by Schaller et al.^29^.

Our results showed that the nuclear membrane protein SUN1/2 requires its N-terminal domain to interact with HIV-1 capsid. This interaction is likely to take place in the nucleus and it is important for the ability of the protein to block HIV-1 infectivity. Interestingly, our experiments demonstrated that the SUN1 block to HIV-1 infection is after the viral core has been transported into the nuclear compartment, which is in agreement with the notion that SUN1 interacts with the HIV-1 core in the nucleus. Previous observations have shown that overexpression of SUN1/2 proteins blocks HIV-1 infection after reverse transcription but before integration^22^. Because reverse transcription occurs in the nuclear compartment the interaction of SUN1 with the HIV-1 core in the nucleus is likely to disrupt the subsequent steps of replication^26–28^. One possibility is that SUN1 binding to capsid sequesters the viral complexes away from critical sites for replication such as open chromatin sites.

Overexpression of SUN1 or SUN2 blocks HIV-1 infection; however, the role of endogenously expressed SUN1 and SUN2 in HIV-1 replication is not clear. For this purpose, we generated knockouts for the expression of SUN1 and/or SUN2 in human haploid HAP-1 cells, and investigated whether HIV-1 infection is affected. To our surprise, complete depletion of SUN1 and/or SUN2 by using CRISPR/Cas9 did not affect HIV-1 replication suggesting that endogenous SUN1 and SUN2 does not have a role in HIV-1 replication of haploid HAP-1 cells. In agreement with our results, knocking out the expression of SUN1 using CRISPR/Cas9 in THP-1 cells did not affect HIV-1 infection^29^. However, depletion of SUN2 using CRIPSR/Cas9 in THP-1 cells mildly affected HIV-1 infection. More recent results, depleting SUN1/2 using siRNA in HEK293T cells dramatically decreased infectivity of HIV-1^30^. To further complicate matters, depletion of SUN2 in human primary T cells affects proliferation and viability^31^. Although several laboratories have attempted depletion of this protein further experiments will be necessary to establish whether endogenous expression of SUN1/2 contribute to HIV-1 infection.

Overall our work showed that HIV-1 restriction by overexpression of SUN1/2 is mediated by the interaction of the N-terminal domain of SUN1/2 with the HIV-1 core occurs in the nuclear compartment.

## Conclusions

This work provides mechanistic understanding on how the overexpression of SUN1/2 proteins block HIV-1 infection in human cells. Our experiments showed that HIV-1 restriction is mediated by the interaction between the N-terminal domain of SUN1/2 proteins and the HIV-1 core in the nuclear compartment.

## Methods

### Cells, viruses and compounds

HT1080 cells (ATCC CCL-121), CHME3 cells (ATCC CRL-3304), and HAP-1 haploid cells (Horizon discovery) were grown in Dulbecco’s modified Eagle’s medium (DMEM), supplemented with 10% fetal calf serum (FCS), 100 IU/mL of penicillin and 100 μg/mL of streptomycin at 37 °C in 5% CO2. Cell were seeded in 24-well plates (50,000 cells/well) 24 h prior to infection with HIV-1-GFP viruses pseudotyped with the VSV-G envelope.

### Generation of stable cell lines

Lentivirus for transduction was produced by transfection of HEK 293 T cells with 3ug VSV-G, 7ug MLV-Gag Pol, and 7ug of plasmids expressing wild type and variant SUN1/2 proteins. Transfections were performed using polyethylenimine (Polysciences) in a 10-cm dish. Viruses were harvested 48 h after transfection, filtered through a 0.45-μm filter (Millipore), and used to transduce HT1080 cells for five days at a ratio of 1:1 with virus and complete media. Transduced cell lines were selected in 0.6 ug/ml of puromycin for 5 days. Stably expressing cells were expanded and kept in 0.6ug/ml of puromycin to maintain selection.

### Western Blot

Cellular proteins were extracted with RIPA buffer (1% NP40, 0.5% Deoxycholate, 0.05% SDS, 1X Protease Inhibitor, 25 mM Tris pH 8, 150 mM NaCl), as previously described^32^. Detection of proteins by Western blotting was performed using anti-FLAG (Sigma) and anti-GAPDH (Sigma) antibodies. Bands were detected and scanned using a Li-Cor Odyssey Imaging System in the 700 channel (original blots are provided in the supplementary file).

### Infection with viruses expressing green fluorescent protein (GFP)

Recombinant human immunodeficiency virus type 1 (HIV-1), simian immunodeficiency virus (SIVmac), B-tropic murine leukemia virus (BMLV), human immunodeficiency virus type 2 (HIV-2), HIV-1, bovine immunodeficiency virus (BIV), equine infectious anemia virus (EIAV), Moloney murine leukemia virus (MMLV), feline immunodeficiency virus (FIV) expressing GFP were prepared as previously described^33^. All recombinant viruses were pseudotyped with the VSV-G glycoprotein. For infections, human HT1080 fibrosarcoma cell line or microglial CHME3 cell line stably expressing SUN1/2 constructs were challenged with increasing amounts of the indicated virus for 24 h. Cells were washed and returned to culture for 48 h, and the percentage of GFP-positive cells was determined by flow cytometry (Becton Dickinson). Viral stocks were titrated by serial dilution on Cf2Th cells to determine the concentration of infectious viruses. As control, cells stably transduced with the empty vector LPCX were challenged with the same viruses.

### Subcellular fractionation to detect HIV-1 capsid in the nucleus

HT1080 cells overexpressed for SUN1-FLAG or LPCX vector control were challenged with HIV-1 viruses at a MOI = 2 for the indicated times. Protocol was used as outlined^26^. Cells were harvested using trypsin and were washed twice with cold PBS by centrifugation at 4 °C. Supernatant was discarded and cell pellet was re-suspended in PBS. A fraction of the cell suspension was centrifuged and cell pellet was re-suspended in lysis buffer and incubated for 1 h on ice then centrifuged for 1 h at 4 °C. The resulting supernatant was mixed with Laemmli buffer and used to measure the total amount of capsid. The remaining aliquot of the cell suspension was pelleted and re-suspended in lysis buffer and incubated on ice. Subsequently, the sample was centrifuged at 4 °C. The resulting supernatant and pellet correspond to cytosolic and nuclear fractions, respectively. Next, a fraction of the supernatant was mixed with Laemmli buffer and used as cytosolic fraction. The nuclear pellet was washed twice using lysis buffer without NP-40 by gently inverting the tube several times. The sample was then centrifuged and the nuclear pellet was re-suspended in extraction buffer, and incubated on ice. Subsequently, the sample was centrifuged at 4 °C. A fraction of the supernatant was mixed with Laemmli buffer and used as nuclear fraction. Proportional amounts of total, cytosolic, and nuclear fractions were analyzed by western blot using anti-p24, anti-Nopp140, anti-a-tubulin, or anti-GAPDH antibodies detailed below.

### Generation of SUN1, SUN2, and SUN1/SUN2 double KO

Hap-1 SUN1, SUN2, and SUN1/SUN2 double knockout (KO) cell lines were generated by using the clustered regularly interspaced short palindromic repeat (CRISPR)-Cas9 gene system. The CRISPR genomic guide RNA sequences for human monoploid fibroblast HAP-1 cells SUN1 CRISPR KO was created using a 1 bp (GRNA sequence: TGTCTCCCTGAAGAACCGAG) deletion in exon 2. CRISPR KO of SUN2 in HAP-1 cells was designed to target exon 4. Sanger sequencing confirmed the 8 bp (GRNA sequence: CTTGCGCCCCACAAGCCCGC) deletion in knockout “3”. The resultant deletions caused a frameshifts that inserted several stop codons in the open reading frame of SUN2. The SUN1/SUN2 CRISPR double knockout generated included a 1 bp (GRNA sequence: TGTCTCCCTGAAGAACCGAG) deletion in exon 2 of SUN1 and 1 bp (GRNA sequence: CTTGCGCCCCACAAGCCCGC) insertion in exon 4 of SUN2.

### Capsid binding assay

HEK293T cells were transiently transfected with LPCX vector expressing mutant or wild type SUN1/2 proteins. After 24 h, cells were lysed in RIPA buffer for 30 min on ice and spun down for 30 min at 4 °C. Lysates were then diluted in capsid binding buffer and incubated with capsid for 1 h at room temperature. Subsequently, samples were layered on top of a sucrose cushion and spun down in a pre-chilled ultracentrifuge for 1 h at 100,000×*g*. Soup (INPUT) and pellet (BOUND) fractions were analyzed by Western blotting using anti-FLAG and anti-p24 antibodies.

### Antibodies

Rabbit Monoclonal clone EPR6557 antibody against SUN2 was obtained from Millipore. Rabbit polyclonal antibody against SUN1 was obtained from Genetex. Anti-FLAG rabbit polyclonal, Anti-GAPDH rabbit polyclonal, anti-mouse Alexa 594, and anti-rabbit Alexa 488 were obtained from Sigma.

### Immunofluorescence

Transfections of cell monolayers were performed using PIE reagent. Transfections were incubated at 37 °C for 24 h. Indirect immunofluorescence microscopy was performed as previously described^34,35^. Transfected monolayers grown on coverslips were washed twice with PBS1X (NaCl 137 mM, KCl 2.7 mM, Na2HPO4·2H2O 10 mM, KH2PO4 mM) and fixed for 15 min in 3.9% paraformaldehyde in PBS1X. Fixed cells were washed twice in PBS1X, permeabilize for 4 min in permeabilizing buffer (0.5% Triton X-100 in PBS), and then blocked in PBS1X containing 2% bovine serum albumin (blocking buffer) for 1 h at room temperature. Cells were then incubated for 1 h at room temperature with M2-Flag primary antibodies diluted in blocking buffer. After three washes with PBS, cells were incubated for 30 min in anti-mouse secondary antibody. Cells were subsequently washed with PBS and stained with 1 μg/ml of 49,69-diamidino-2-phenylindole (DAPI). Samples were mounted for fluorescence microscopy by using the ProLong Antifade Kit (Molecular Probes, Eugene, OR). Images were obtained with a Zeiss Observer.Z1 microscope using a 63 × objective, and deconvolution was performed using the software AxioVision V4.8.1.0 (Carl Zeiss Imaging Solutions).

## Supplementary Information

Supplementary Information.

## Data Availability

Source data available on request.

## Abbreviations

SUN1: Sad1 and UNC 84A
SUN2: Sad1 and UNC 84B
SUN1/2: SUN1 and SUN2
HIV-1-GFP: HIV-1 pseudotyped with VSVG expressing GFP
SIVmac: Simian immunodeficiency virus from macaque monkeys
FIV: Feline immunodeficiency virus
BIV: Bovine immunodeficiency virus
EIAV: Equine infectious anemia virus
B-MLV: B-Tropic murine leukemia virus
HAP-1: Huntington associated protein 1; haploid cells; human
HT1080: Human fibrosarcoma cell line; human
PF74: PF-3450074
PS: Perinuclear staining
NP: Non-perinuclear localization
