# Supplementary material for: Nuclear restriction of HIV-1 infection by SUN1
Source: Sci Rep. 2021 Sep 27;11:19128. doi: 10.1038/s41598-021-98541-4 (PMC8476499; doi:10.1038/s41598-021-98541-4)
Supplement: Supplementary file 1 — Supplementary Information. [file 41598_2021_98541_MOESM1_ESM.pdf]

Figure 2B

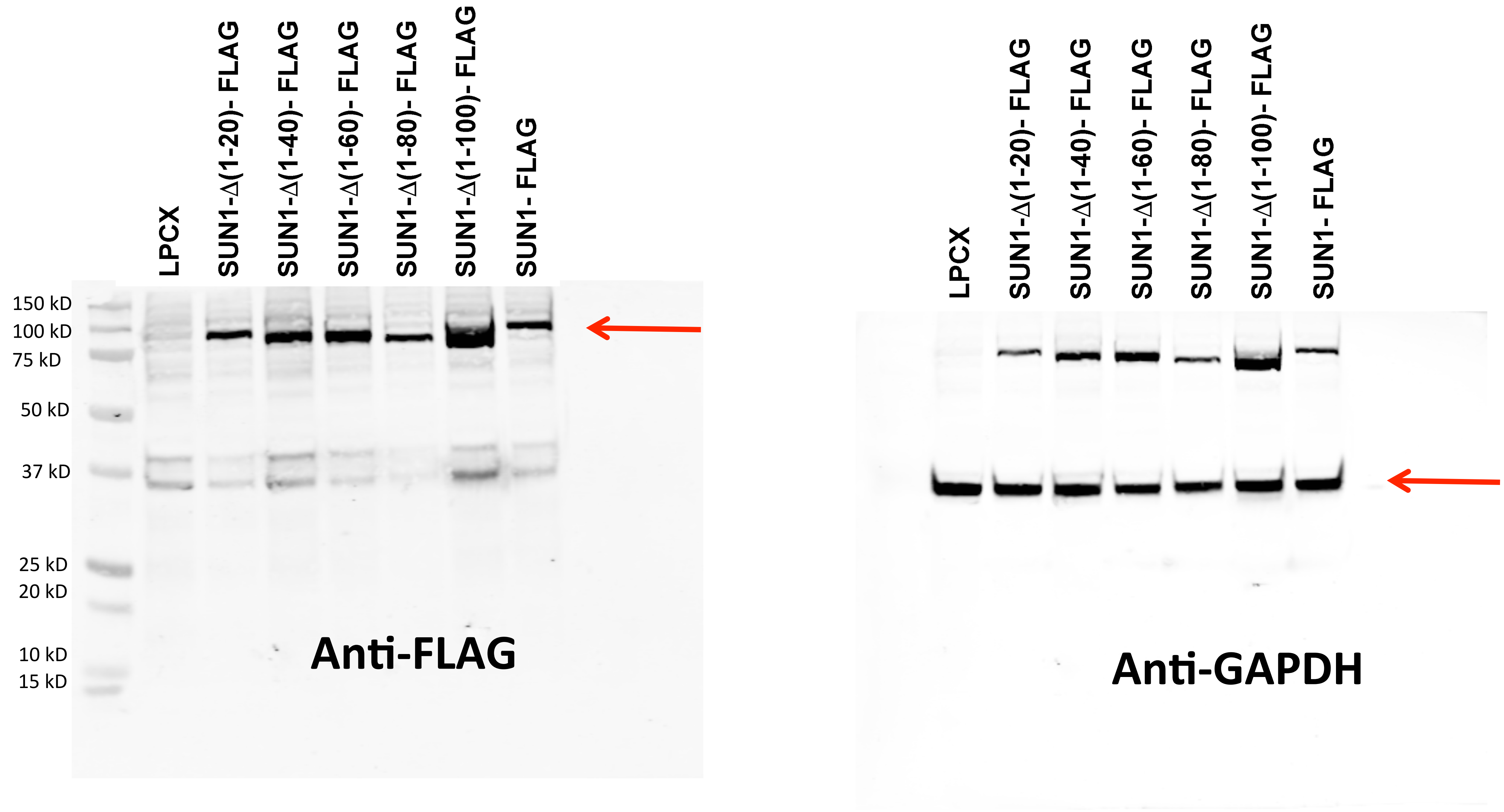
LPCX
SUN1-Δ(1-20)- FLAG
SUN1-Δ(1-40)- FLAG
SUN1-Δ(1-60)- FLAG
SUN1-Δ(1-80)- FLAG
SUN1-Δ(1-100)- FLAG
SUN1- FLAG
150 kD
100 kD
75 kD
50 kD
37 kD
25 kD
20 kD
10 kD
15 kD
Anti-FLAG
LPCX
SUN1-Δ(1-20)- FLAG
SUN1-Δ(1-40)- FLAG
SUN1-Δ(1-60)- FLAG
SUN1-Δ(1-80)- FLAG
SUN1-Δ(1-100)- FLAG
SUN1- FLAG
Anti-GAPDH

Figure 3

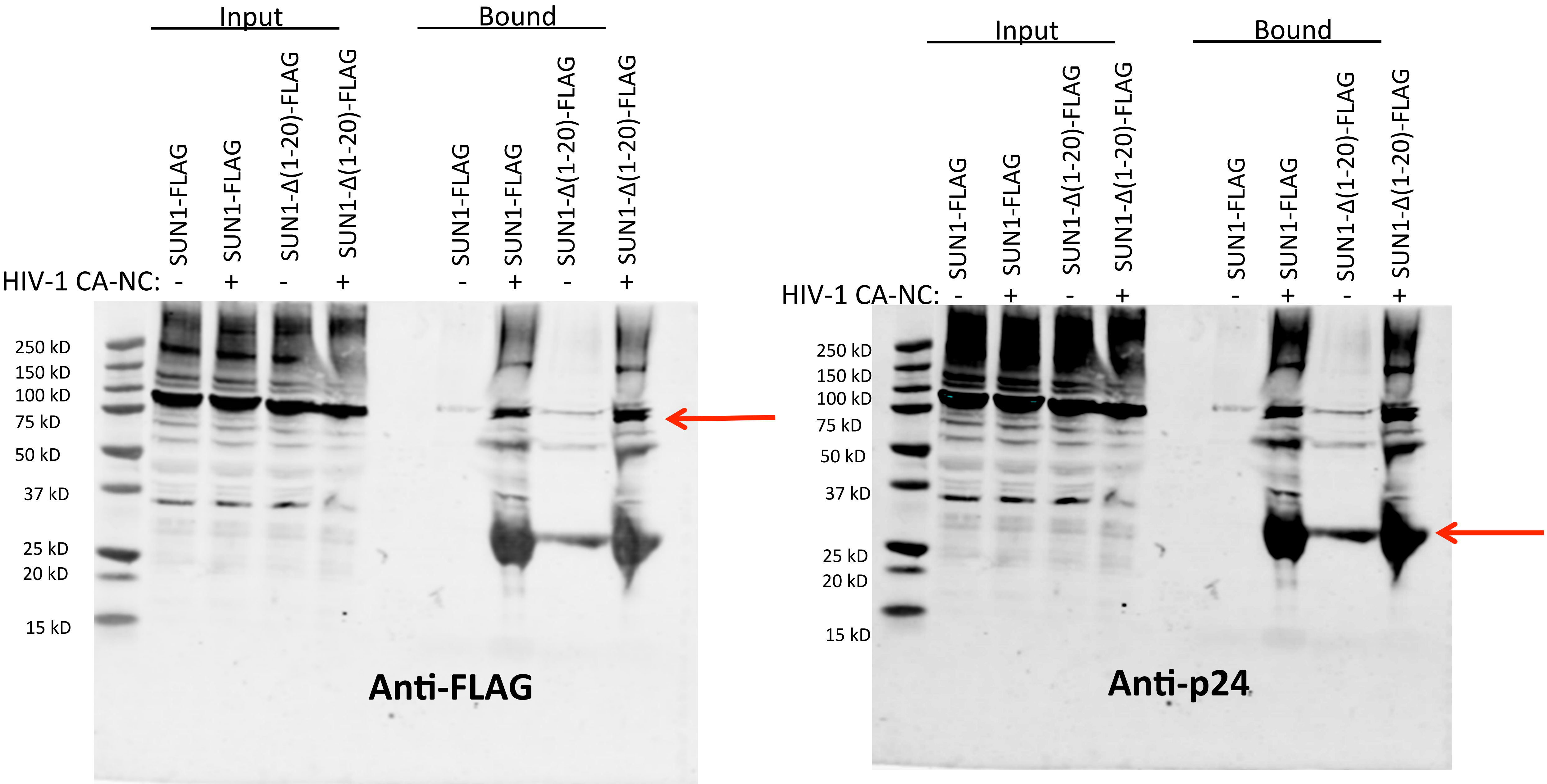

Input
Bound
SUN1-FLAG
SUN1-FLAG
SUN1-Δ(1-20)-FLAG
SUN1-Δ(1-20)-FLAG
SUN1-FLAG
SUN1-FLAG
SUN1-Δ(1-20)-FLAG
SUN1-Δ(1-20)-FLAG
HIV-1 CA-NC: - + - + - + - +
250 kD
150 kD
100 kD
75 kD
50 kD
37 kD
25 kD
20 kD
15 kD
Anti-FLAG
Input
Bound
SUN1-FLAG
SUN1-FLAG
SUN1-Δ(1-20)-FLAG
SUN1-Δ(1-20)-FLAG
SUN1-FLAG
SUN1-FLAG
SUN1-Δ(1-20)-FLAG
SUN1-Δ(1-20)-FLAG
HIV-1 CA-NC: - + - + - + - +
250 kD
150 kD
100 kD
75 kD
50 kD
37 kD
25 kD
20 kD
15 kD
Anti-p24

Figure 3

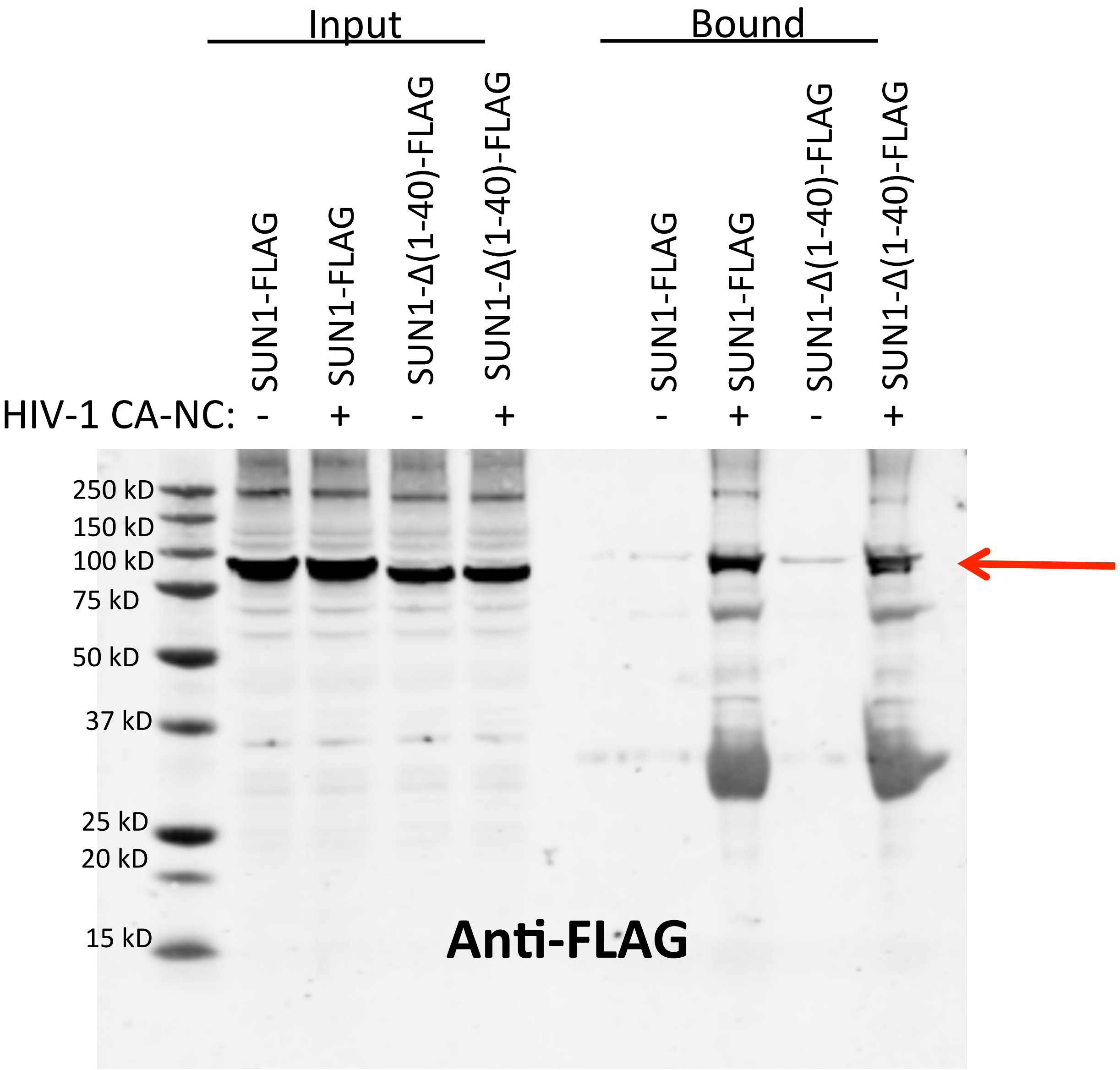

Input
Bound
SUN1-FLAG
SUN1-FLAG
SUN1-Δ(1-40)-FLAG
SUN1-Δ(1-40)-FLAG
SUN1-FLAG
SUN1-FLAG
SUN1-Δ(1-40)-FLAG
SUN1-Δ(1-40)-FLAG
HIV-1 CA-NC: - + - + - + - +
250 kD
150 kD
100 kD
75 kD
50 kD
37 kD
25 kD
20 kD
15 kD
Anti-FLAG

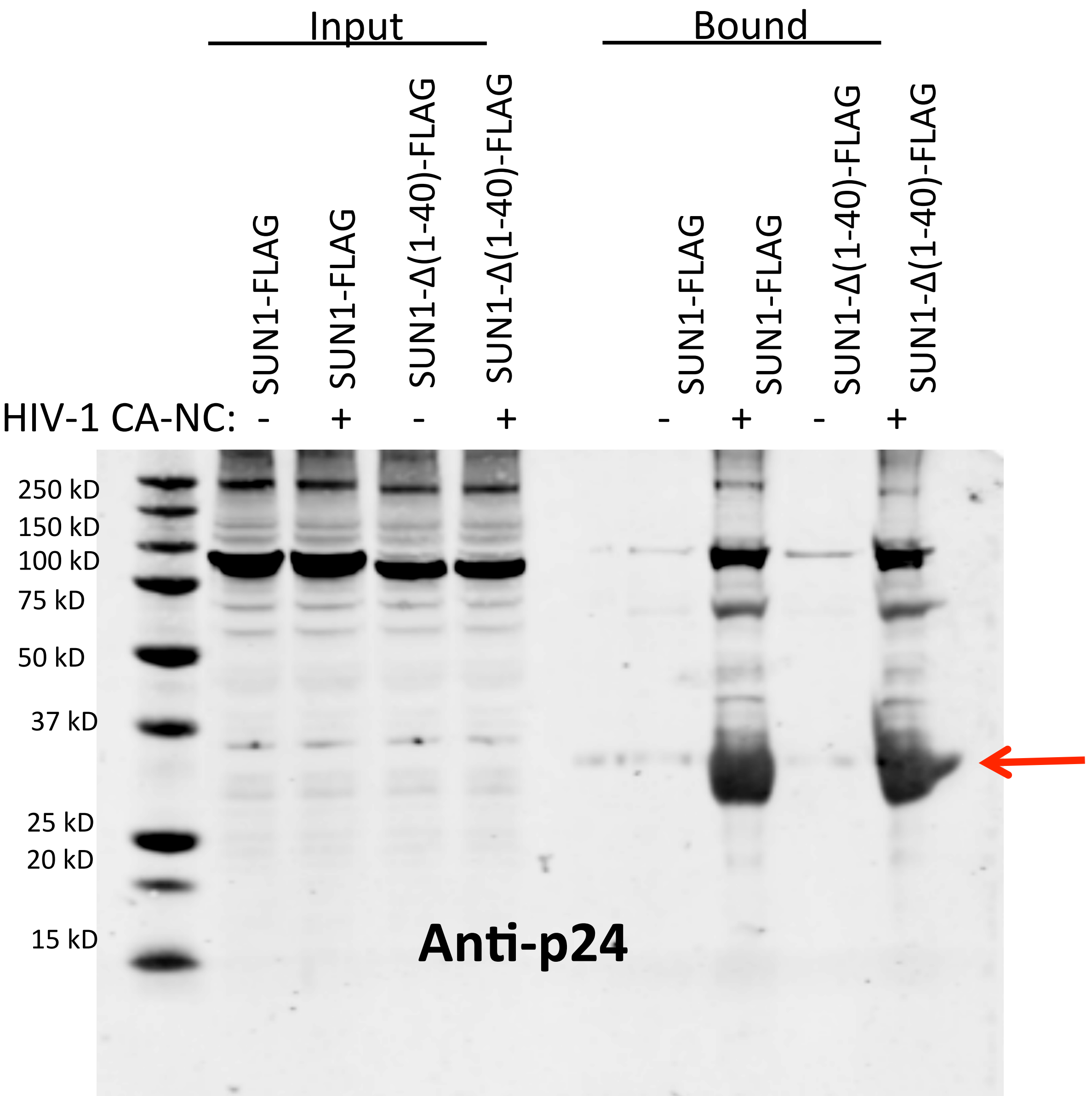

Input
Bound
SUN1-FLAG
SUN1-FLAG
SUN1-Δ(1-40)-FLAG
SUN1-Δ(1-40)-FLAG
SUN1-FLAG
SUN1-FLAG
SUN1-Δ(1-40)-FLAG
SUN1-Δ(1-40)-FLAG
HIV-1 CA-NC: - + - + - + - +
250 kD
150 kD
100 kD
75 kD
50 kD
37 kD
25 kD
20 kD
15 kD
Anti-p24

Figure 3

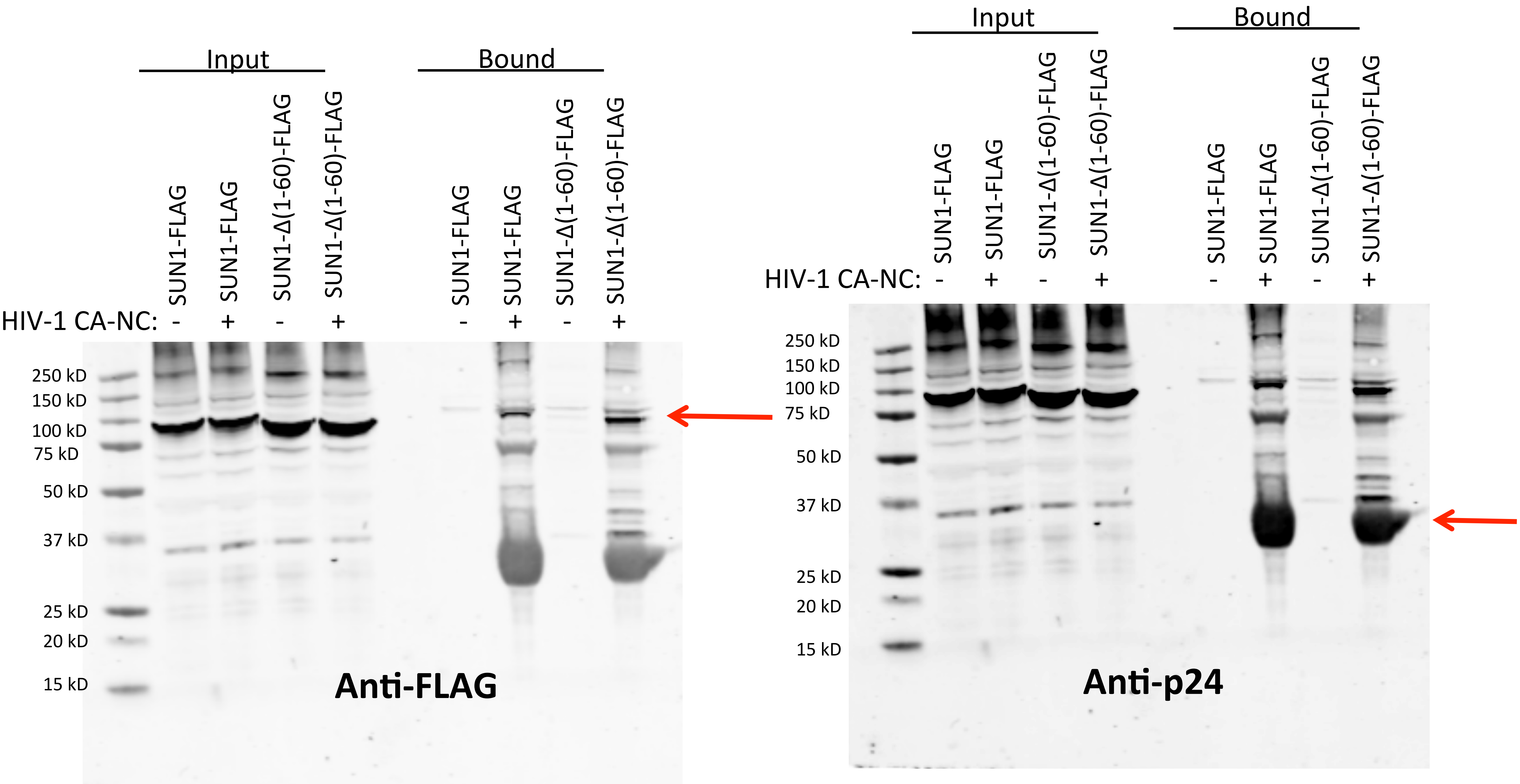

Input
Bound
SUN1-FLAG
SUN1-FLAG
SUN1-Δ(1-60)-FLAG
SUN1-Δ(1-60)-FLAG
SUN1-FLAG
SUN1-FLAG
SUN1-Δ(1-60)-FLAG
SUN1-Δ(1-60)-FLAG
HIV-1 CA-NC: - + - + - + - +
250 kD
150 kD
100 kD
75 kD
50 kD
37 kD
25 kD
20 kD
15 kD
Anti-FLAG
Input
Bound
SUN1-FLAG
SUN1-FLAG
SUN1-Δ(1-60)-FLAG
SUN1-Δ(1-60)-FLAG
SUN1-FLAG
SUN1-FLAG
SUN1-Δ(1-60)-FLAG
SUN1-Δ(1-60)-FLAG
HIV-1 CA-NC: - + - + - + - +
250 kD
150 kD
100 kD
75 kD
50 kD
37 kD
25 kD
20 kD
15 kD
Anti-p24

Figure 3

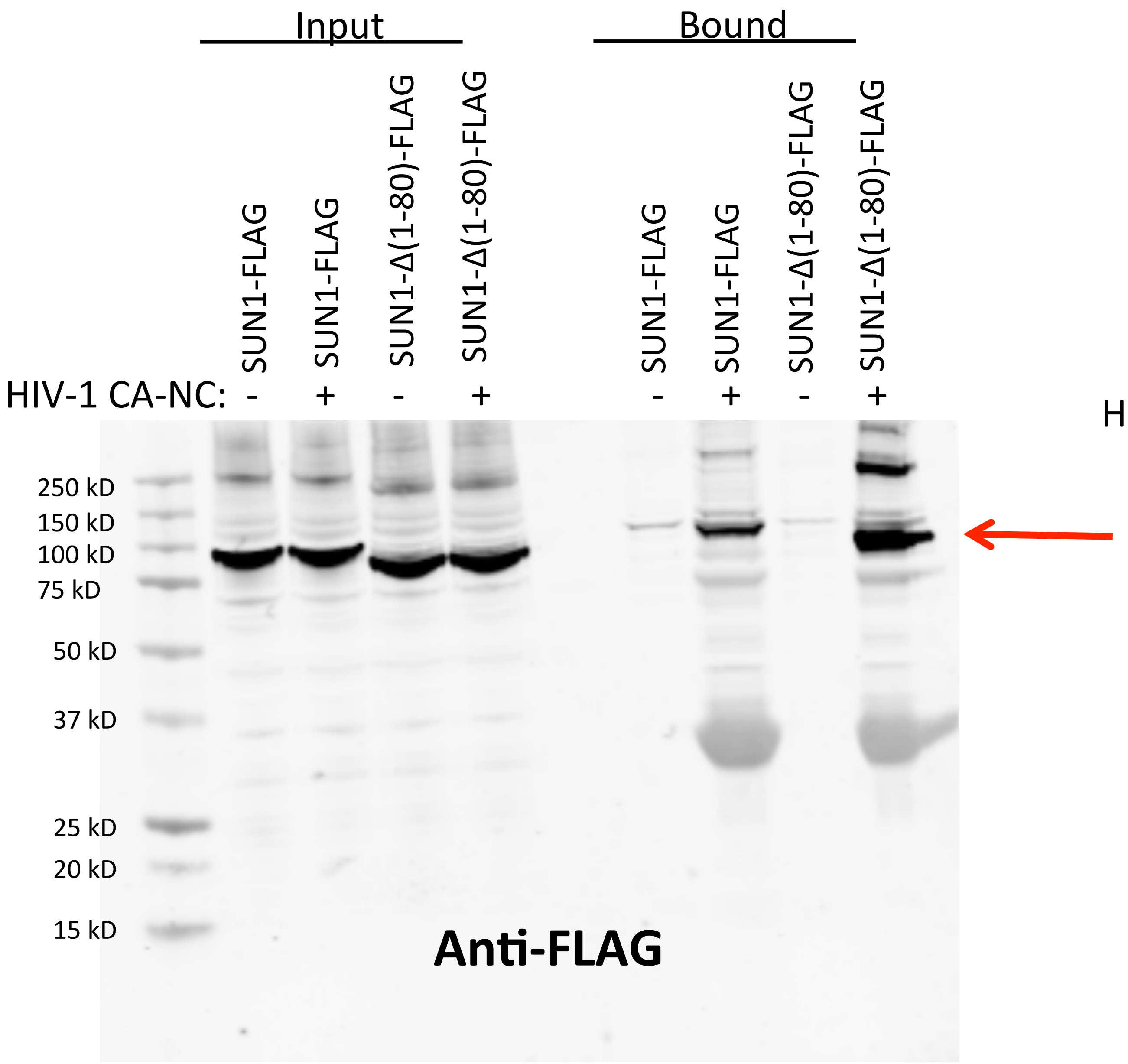

Input
Bound
SUN1-FLAG
SUN1-FLAG
SUN1-Δ(1-80)-FLAG
SUN1-Δ(1-80)-FLAG
SUN1-FLAG
SUN1-FLAG
SUN1-Δ(1-80)-FLAG
SUN1-Δ(1-80)-FLAG
HIV-1 CA-NC: - + - + - + - +
250 kD
150 kD
100 kD
75 kD
50 kD
37 kD
25 kD
20 kD
15 kD
Anti-FLAG

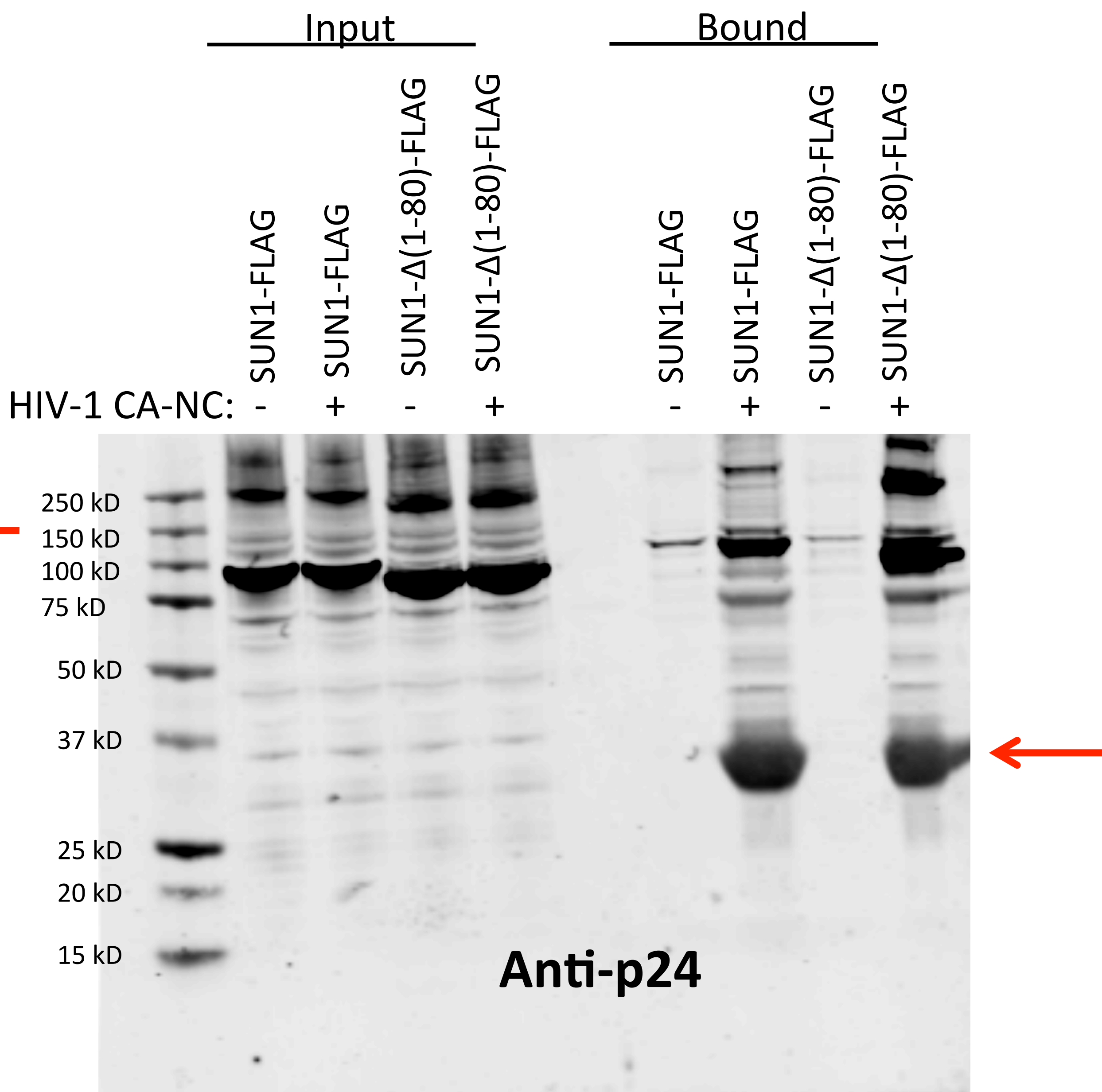

Input
Bound
SUN1-FLAG
SUN1-FLAG
SUN1-Δ(1-80)-FLAG
SUN1-Δ(1-80)-FLAG
SUN1-FLAG
SUN1-FLAG
SUN1-Δ(1-80)-FLAG
SUN1-Δ(1-80)-FLAG
HIV-1 CA-NC: - + - + - + - +
250 kD
150 kD
100 kD
75 kD
50 kD
37 kD
25 kD
20 kD
15 kD
Anti-p24

Figure 3

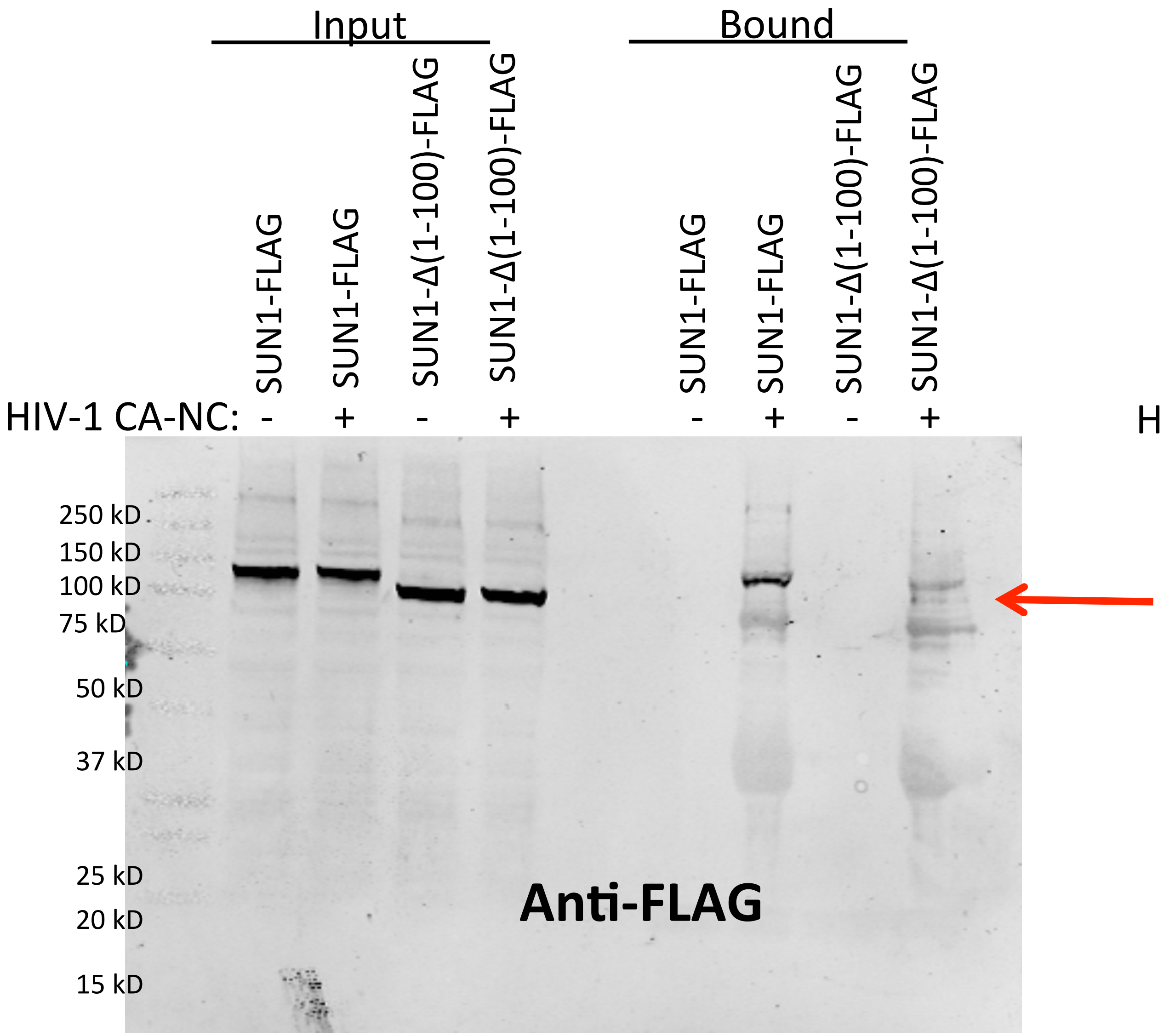

Input
Bound
SUN1-FLAG
SUN1-FLAG
SUN1-Δ(1-100)-FLAG
SUN1-Δ(1-100)-FLAG
SUN1-FLAG
SUN1-FLAG
SUN1-Δ(1-100)-FLAG
SUN1-Δ(1-100)-FLAG
HIV-1 CA-NC: - + - + - + - +
250 kD
150 kD
100 kD
75 kD
50 kD
37 kD
25 kD
20 kD
15 kD
Anti-FLAG

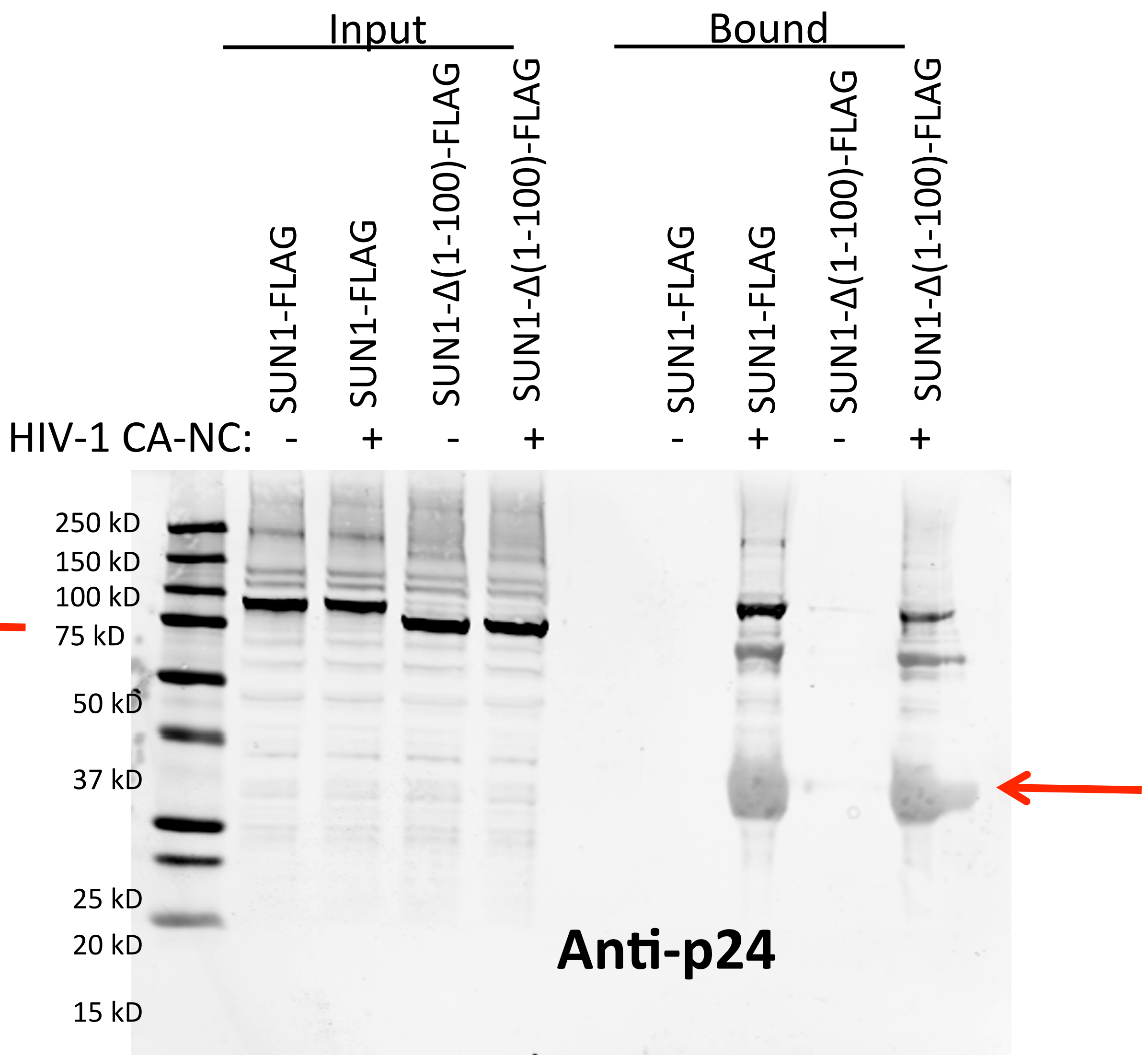

Input
Bound
SUN1-FLAG
SUN1-FLAG
SUN1-Δ(1-100)-FLAG
SUN1-Δ(1-100)-FLAG
SUN1-FLAG
SUN1-FLAG
SUN1-Δ(1-100)-FLAG
SUN1-Δ(1-100)-FLAG
HIV-1 CA-NC: - + - + - + - +
250 kD
150 kD
100 kD
75 kD
50 kD
37 kD
25 kD
20 kD
15 kD
Anti-p24

Figure 5B

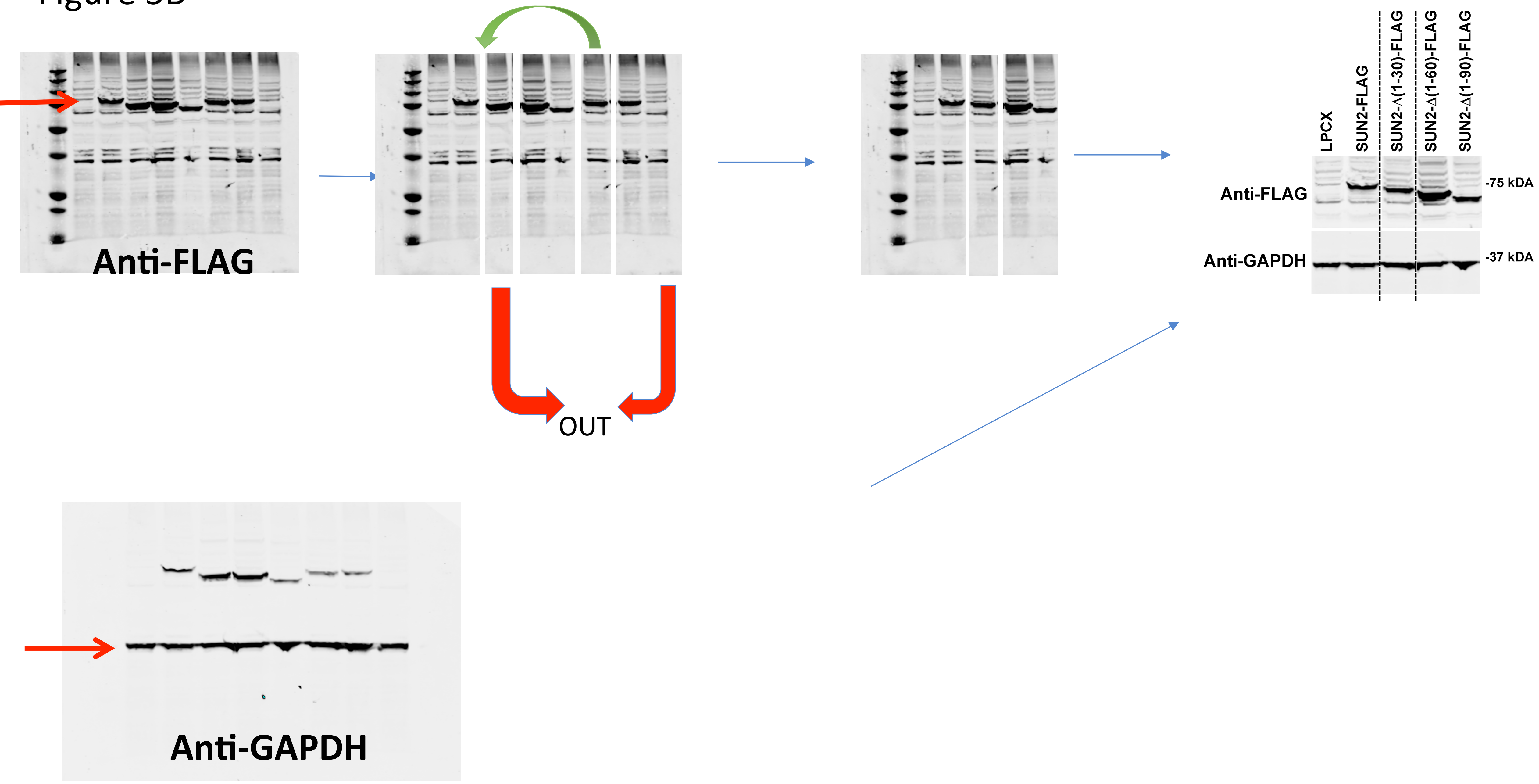

Anti-FLAG
OUT
LPCX
SUN2-FLAG
SUN2-Δ(1-30)-FLAG
SUN2-Δ(1-60)-FLAG
SUN2-Δ(1-90)-FLAG
Anti-FLAG
Anti-GAPDH
-75 kDA
-37 kDA
Anti-GAPDH

Figure 6

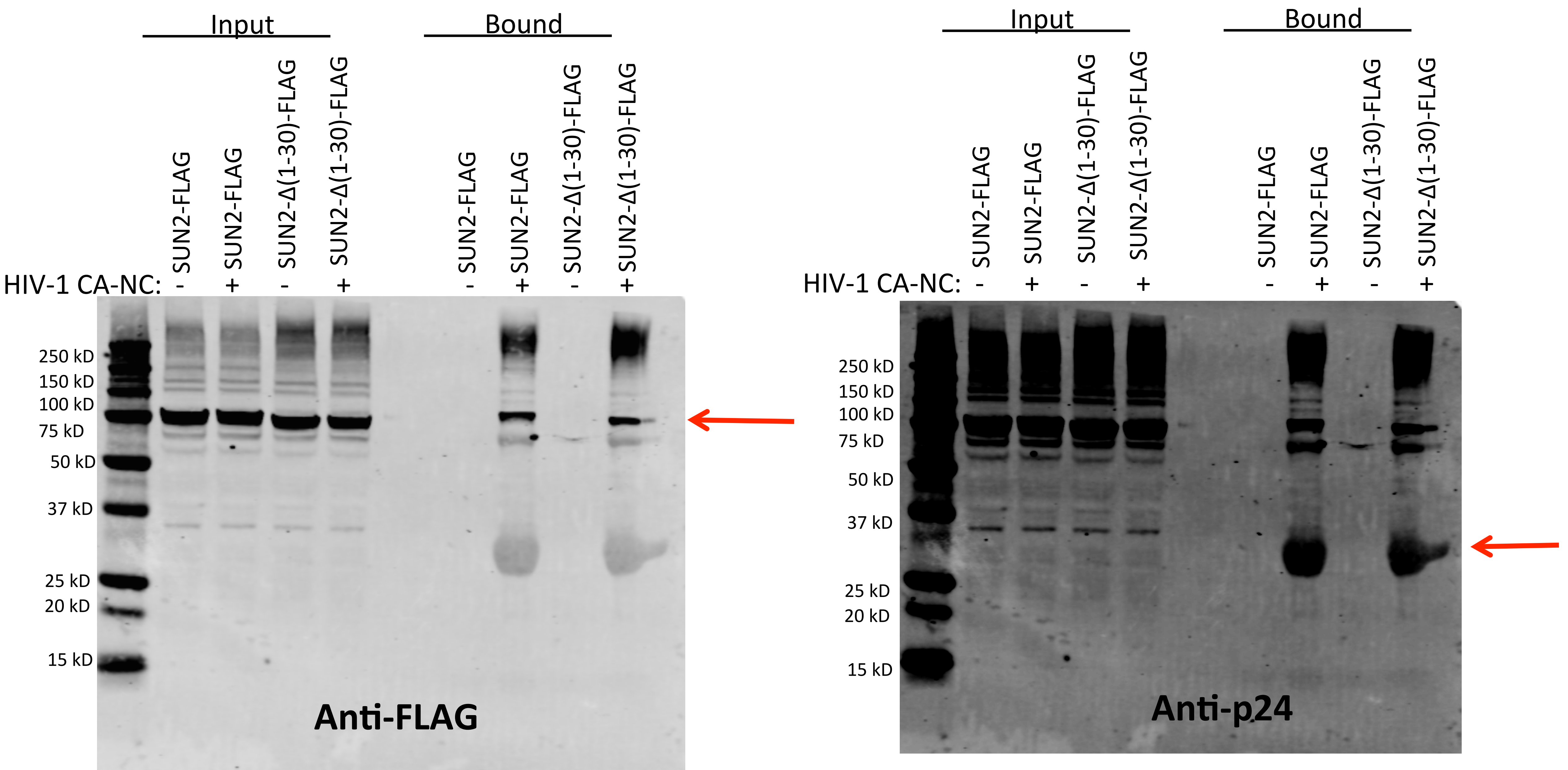

Input
Bound
SUN2-FLAG
SUN2-FLAG
SUN2-Δ(1-30)-FLAG
SUN2-Δ(1-30)-FLAG
SUN2-FLAG
SUN2-FLAG
SUN2-Δ(1-30)-FLAG
SUN2-Δ(1-30)-FLAG
HIV-1 CA-NC: - + - + - + - +
250 kD
150 kD
100 kD
75 kD
50 kD
37 kD
25 kD
20 kD
15 kD
Anti-FLAG
Input
Bound
SUN2-FLAG
SUN2-FLAG
SUN2-Δ(1-30)-FLAG
SUN2-Δ(1-30)-FLAG
SUN2-FLAG
SUN2-FLAG
SUN2-Δ(1-30)-FLAG
SUN2-Δ(1-30)-FLAG
HIV-1 CA-NC: - + - + - + - +
250 kD
150 kD
100 kD
75 kD
50 kD
37 kD
25 kD
20 kD
15 kD
Anti-p24

Figure 6

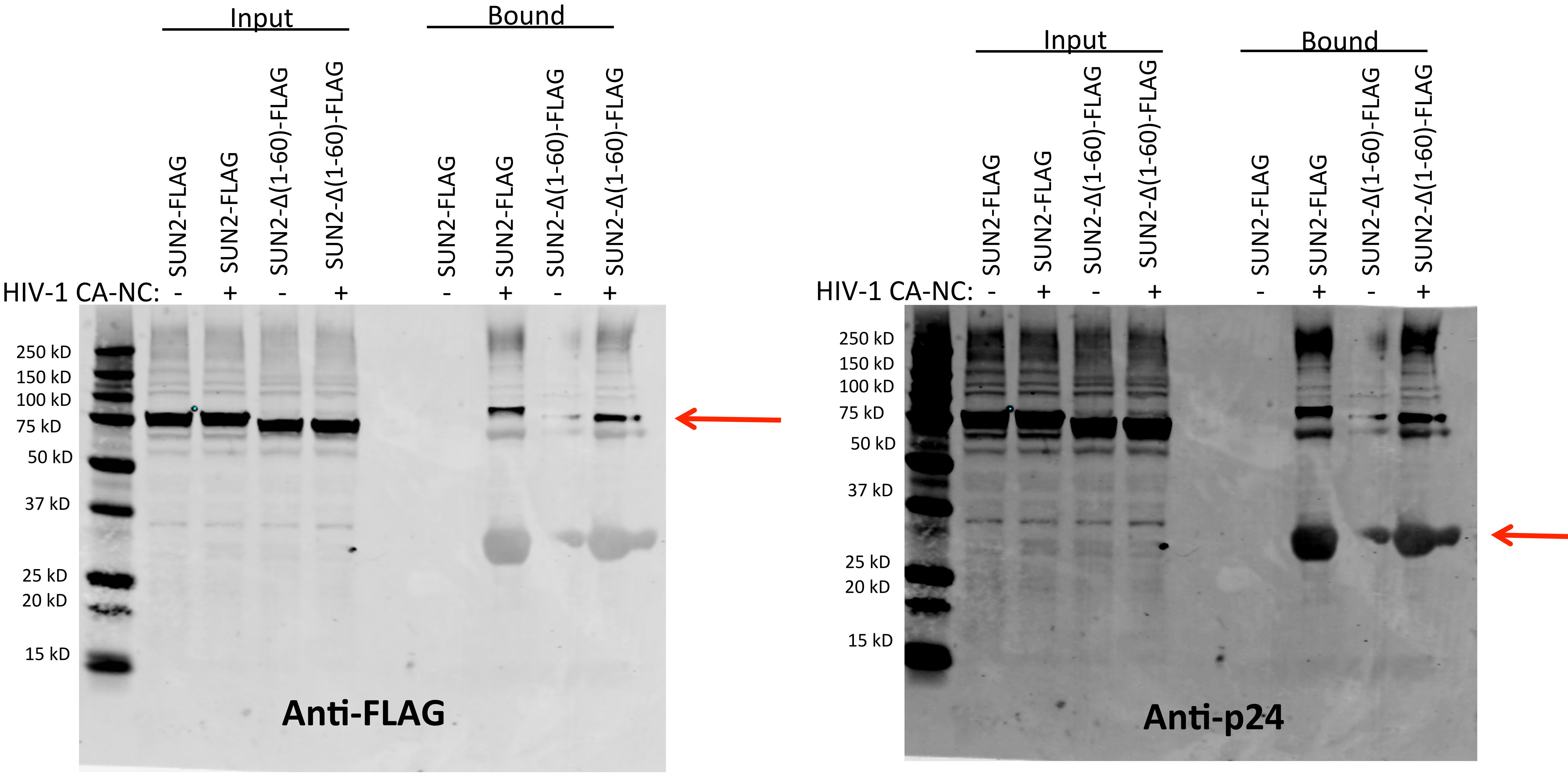

Input
Bound
SUN2-FLAG
SUN2-FLAG
SUN2-Δ(1-60)-FLAG
SUN2-Δ(1-60)-FLAG
SUN2-FLAG
SUN2-FLAG
SUN2-Δ(1-60)-FLAG
SUN2-Δ(1-60)-FLAG
HIV-1 CA-NC: - + - + - + - +
250 kD
150 kD
100 kD
75 kD
50 kD
37 kD
25 kD
20 kD
15 kD
Anti-FLAG
Input
Bound
SUN2-FLAG
SUN2-FLAG
SUN2-Δ(1-60)-FLAG
SUN2-Δ(1-60)-FLAG
SUN2-FLAG
SUN2-FLAG
SUN2-Δ(1-60)-FLAG
SUN2-Δ(1-60)-FLAG
HIV-1 CA-NC: - + - + - + - +
250 kD
150 kD
100 kD
75 kD
50 kD
37 kD
25 kD
20 kD
15 kD
Anti-p24

Figure 6

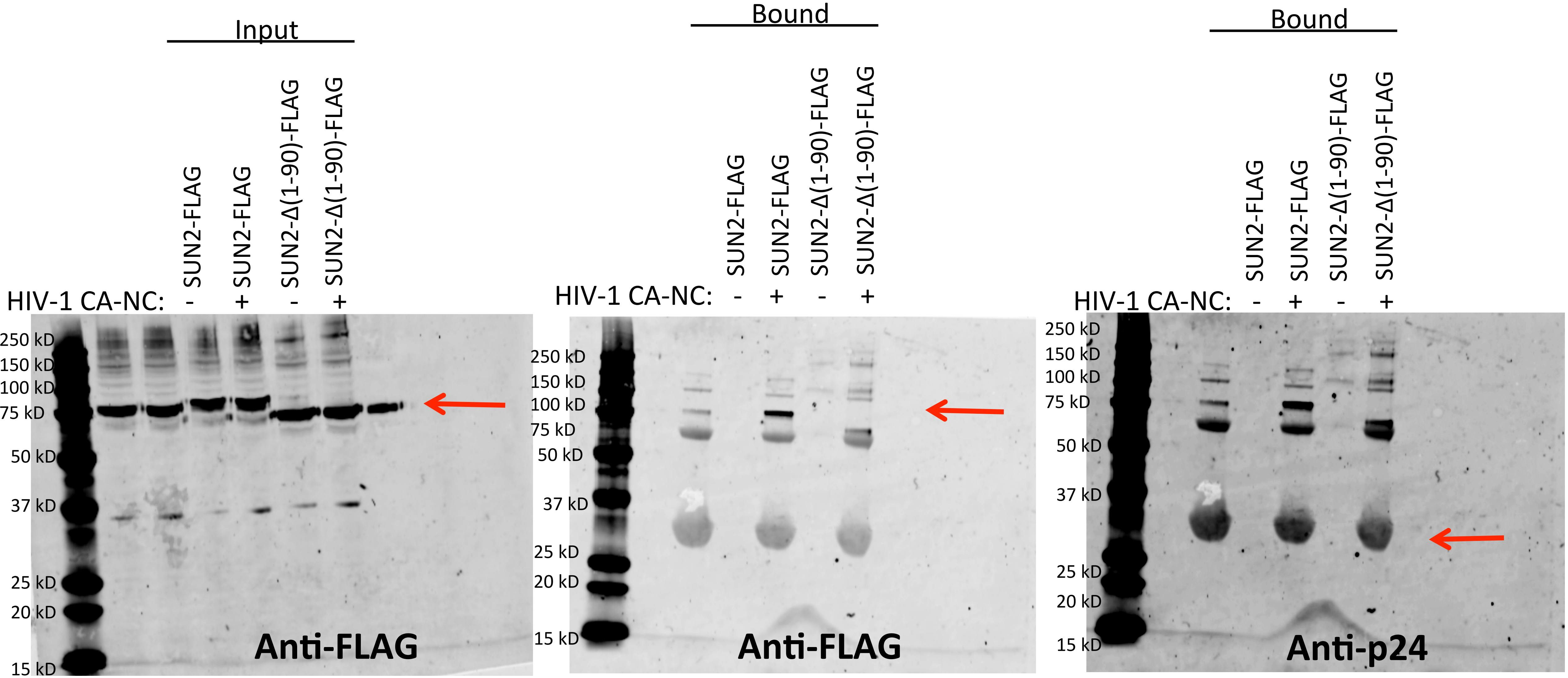

Input
SUN2-FLAG
SUN2-FLAG
SUN2-Δ(1-90)-FLAG
SUN2-Δ(1-90)-FLAG
HIV-1 CA-NC: - + - +
250 kD
150 kD
100 kD
75 kD
50 kD
37 kD
25 kD
20 kD
15 kD
Anti-FLAG
Bound
SUN2-FLAG
SUN2-FLAG
SUN2-Δ(1-90)-FLAG
SUN2-Δ(1-90)-FLAG
HIV-1 CA-NC: - + - +
250 kD
150 kD
100 kD
75 kD
50 kD
37 kD
25 kD
20 kD
15 kD
Anti-FLAG
Bound
SUN2-FLAG
SUN2-FLAG
SUN2-Δ(1-90)-FLAG
SUN2-Δ(1-90)-FLAG
HIV-1 CA-NC: - + - +
250 kD
150 kD
100 kD
75 kD
50 kD
37 kD
25 kD
20 kD
15 kD
Anti-p24

Figure 7A

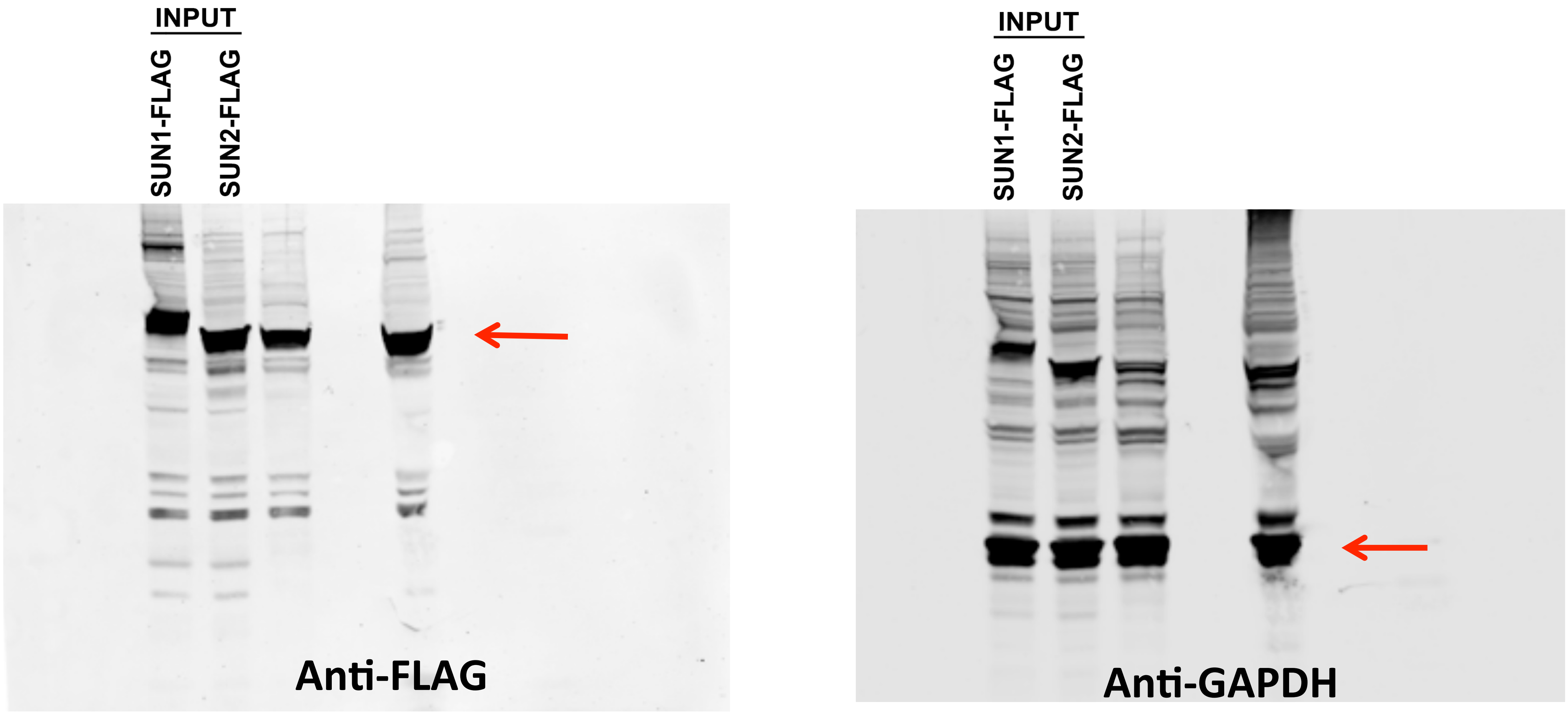
INPUT
SUN1-FLAG
SUN2-FLAG
Anti-FLAG
INPUT
SUN1-FLAG
SUN2-FLAG
Anti-GAPDH

Figure 7A

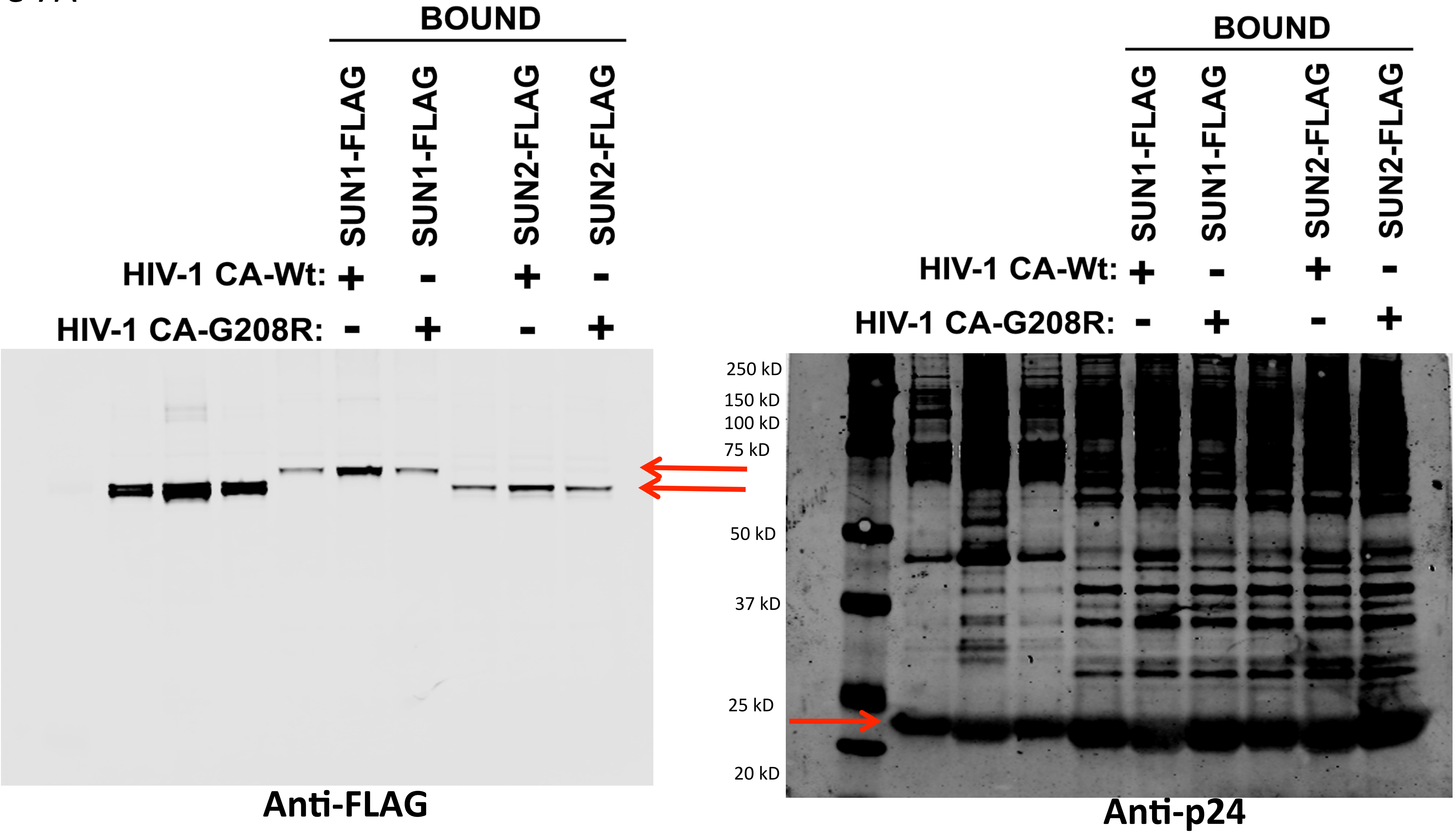
BOUND
SUN1-FLAG
SUN1-FLAG
SUN2-FLAG
SUN2-FLAG
HIV-1 CA-Wt: + - + -
HIV-1 CA-G208R: - + - +
Anti-FLAG
BOUND
SUN1-FLAG
SUN1-FLAG
SUN2-FLAG
SUN2-FLAG
HIV-1 CA-Wt: + - + -
HIV-1 CA-G208R: - + - +
250 kD
150 kD
100 kD
75 kD
50 kD
37 kD
25 kD
20 kD
Anti-p24

Figure 7A

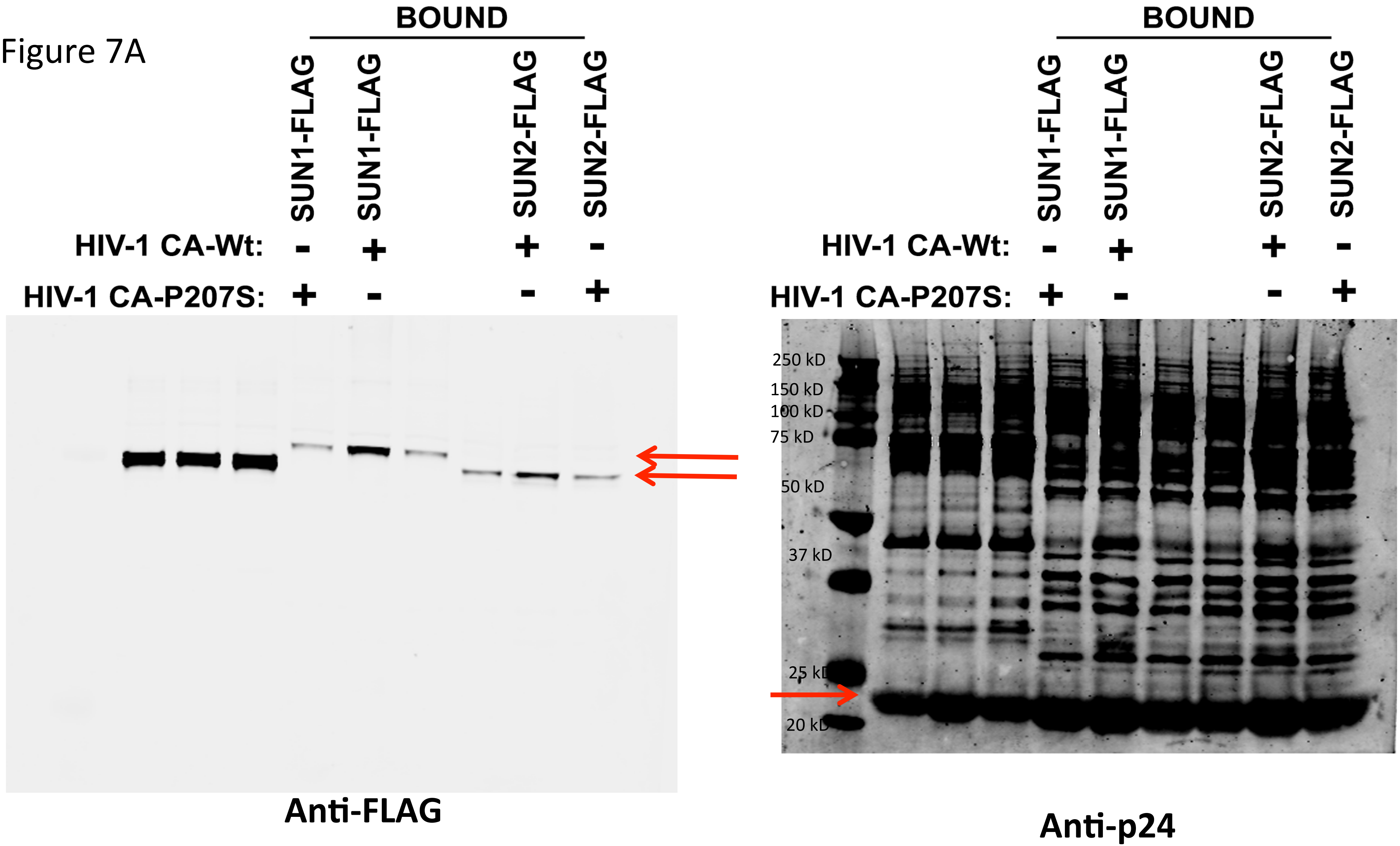
BOUND
SUN1-FLAG
SUN1-FLAG
SUN2-FLAG
SUN2-FLAG
HIV-1 CA-Wt: - + + -
HIV-1 CA-P207S: + - - +
Anti-FLAG
BOUND
SUN1-FLAG
SUN1-FLAG
SUN2-FLAG
SUN2-FLAG
HIV-1 CA-Wt: - + + -
HIV-1 CA-P207S: + - - +
250 kD
150 kD
100 kD
75 kD
50 kD
37 kD
25 kD
20 kD
Anti-p24

Figure 7B

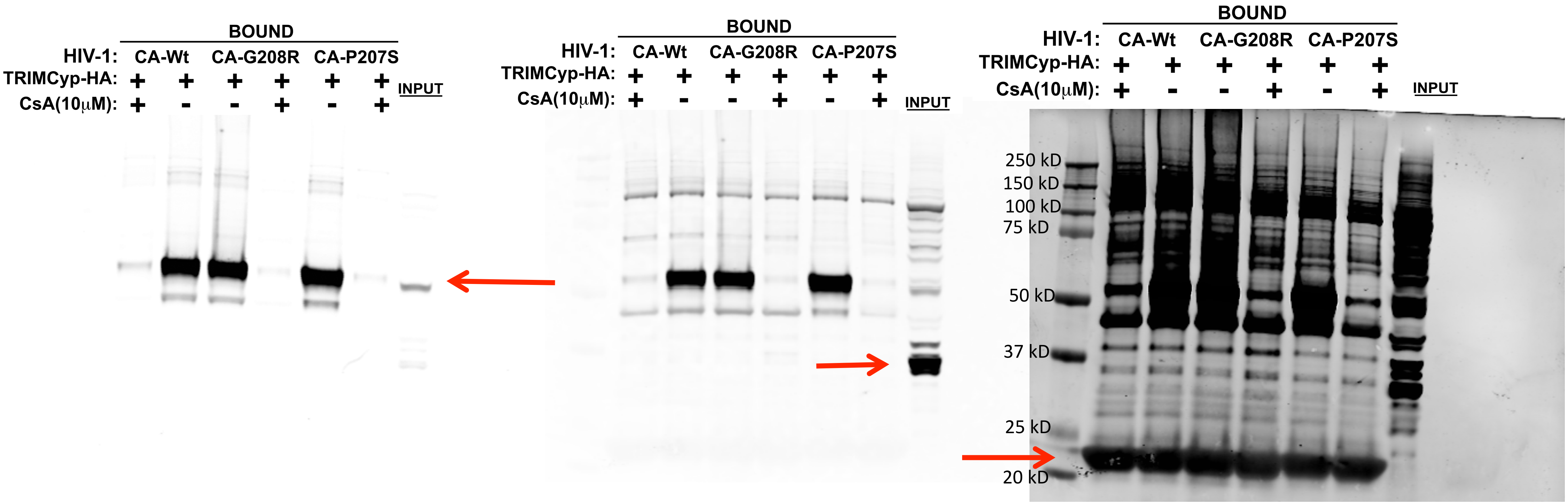

Anti-HA Anti-GAPDH Anti-p24

Figure 9

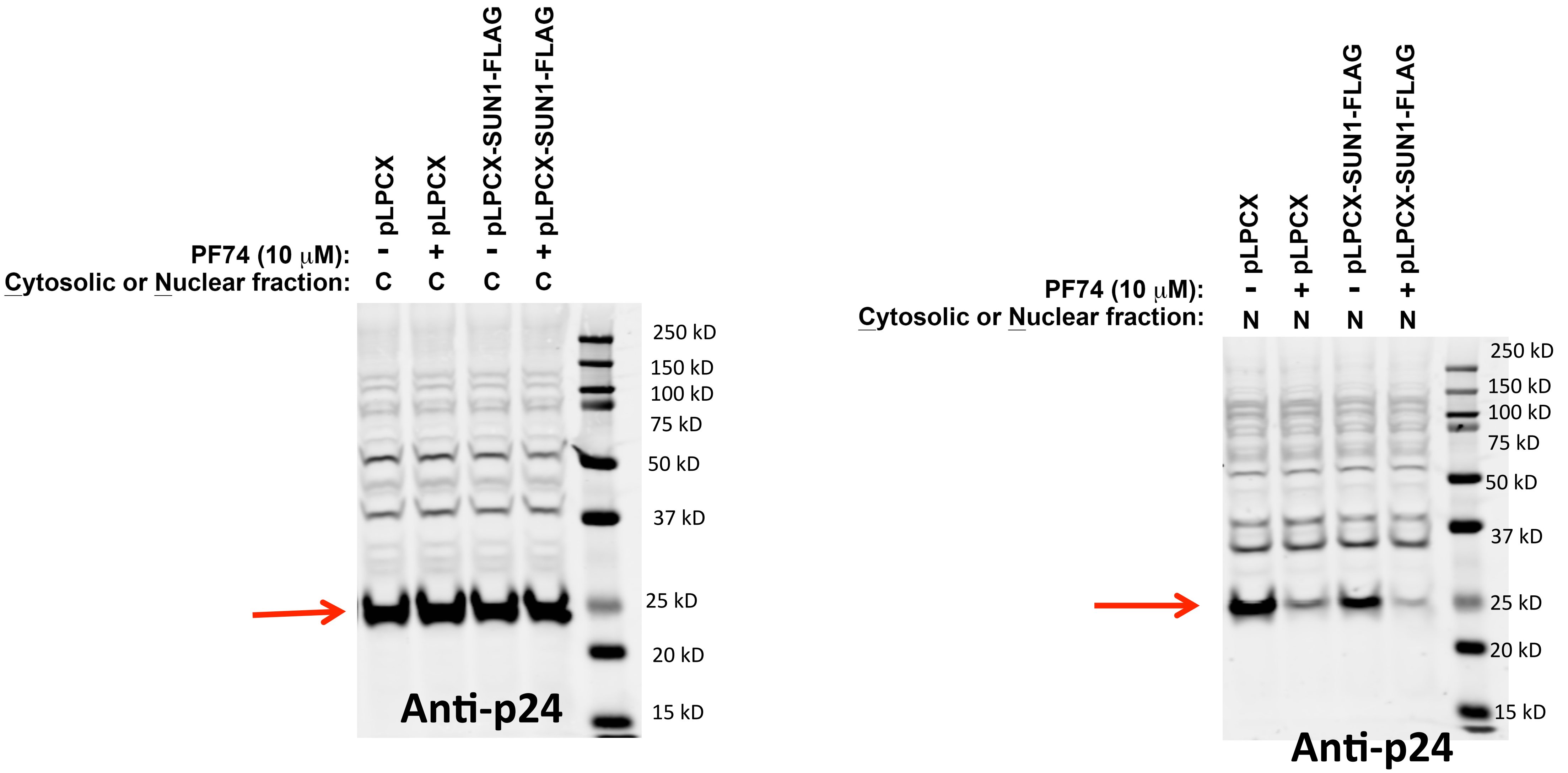

pLPCX
pLPCX
pLPCX-SUN1-FLAG
pLPCX-SUN1-FLAG
PF74 (10 μM): - + - +
Cytosolic or Nuclear fraction: C C C C
250 kD
150 kD
100 kD
75 kD
50 kD
37 kD
25 kD
20 kD
15 kD
Anti-p24
pLPCX
pLPCX
pLPCX-SUN1-FLAG
pLPCX-SUN1-FLAG
PF74 (10 μM): - + - +
Cytosolic or Nuclear fraction: N N N N
250 kD
150 kD
100 kD
75 kD
50 kD
37 kD
25 kD
20 kD
15 kD
Anti-p24

Figure 9

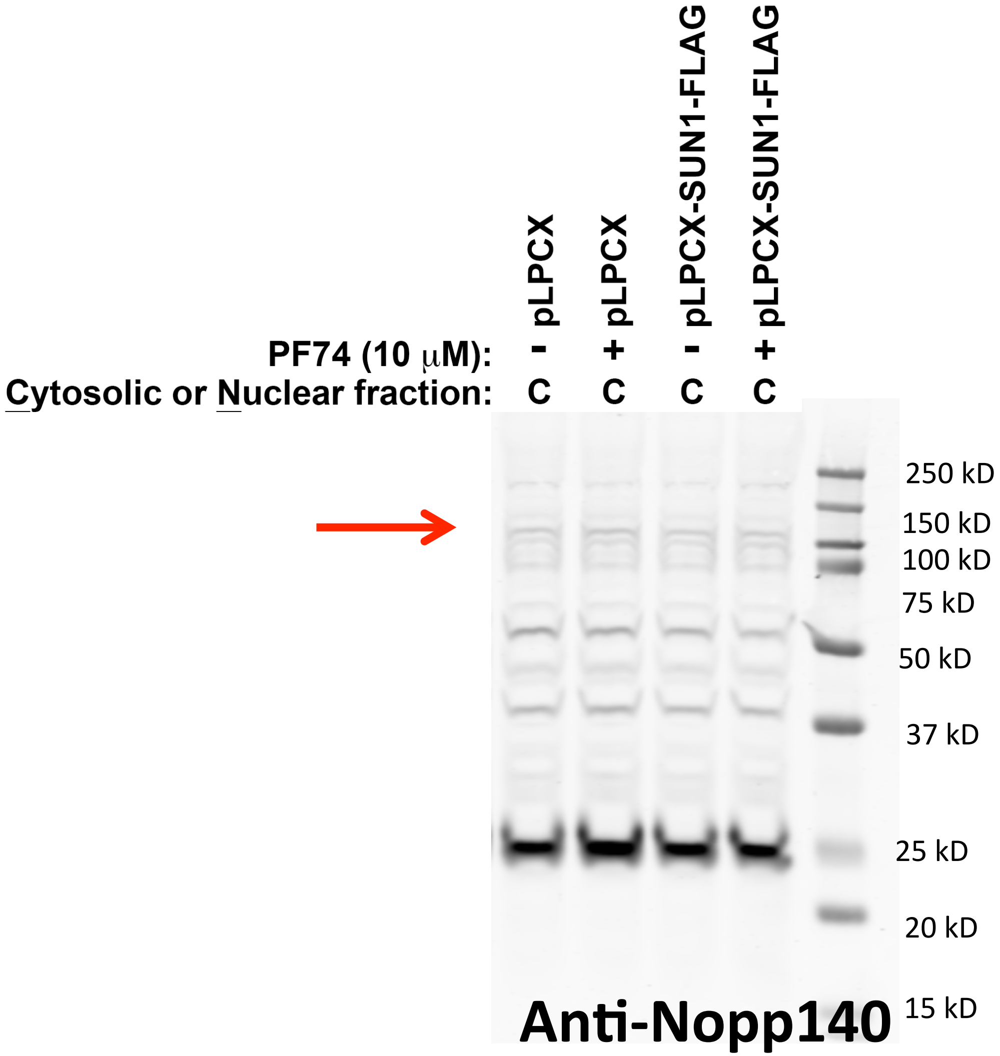
PF74 (10 μM):
Cytosolic or Nuclear fraction:
- pLPCX
+ pLPCX
- pLPCX-SUN1-FLAG
+ pLPCX-SUN1-FLAG
C
C
C
C
250 kD
150 kD
100 kD
75 kD
50 kD
37 kD
25 kD
20 kD
15 kD
Anti-Nopp140

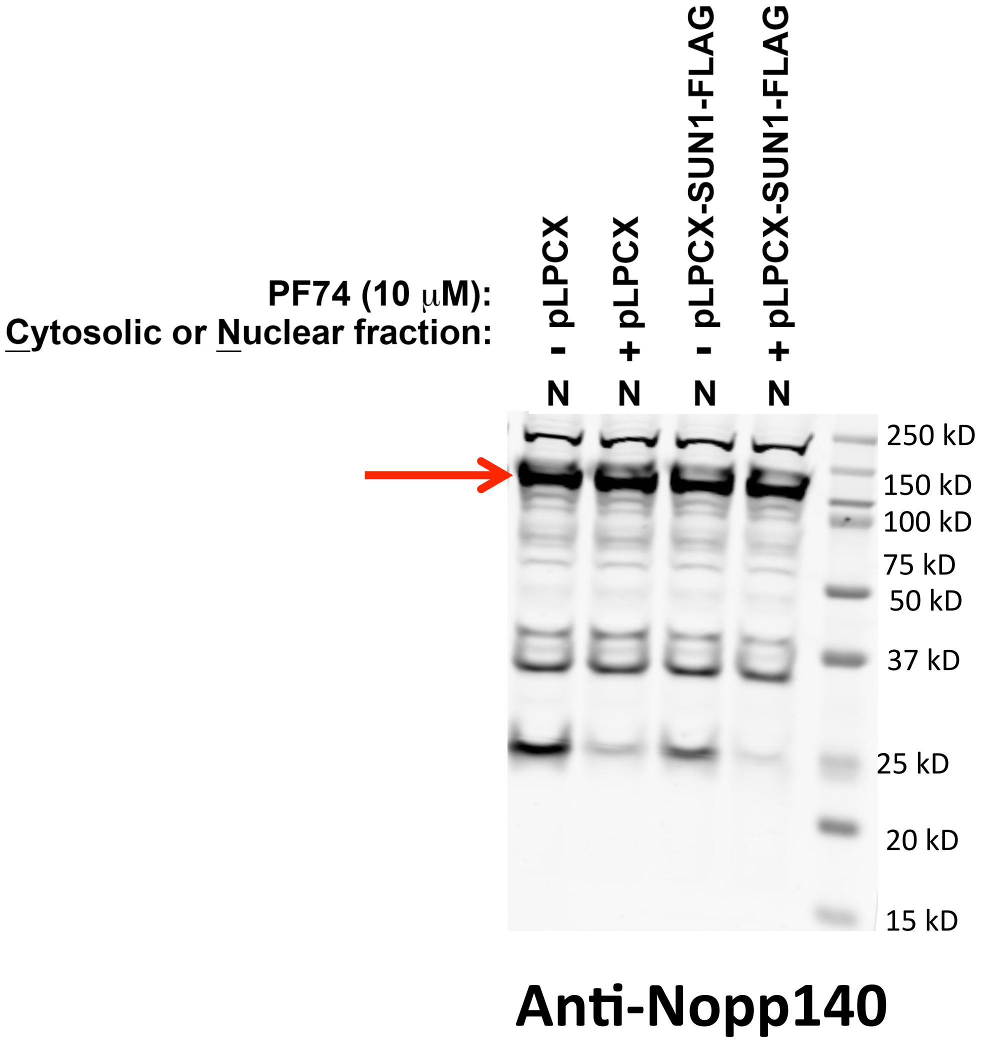
PF74 (10 μM):
Cytosolic or Nuclear fraction:
- pLPCX
+ pLPCX
- pLPCX-SUN1-FLAG
+ pLPCX-SUN1-FLAG
N
N
N
N
250 kD
150 kD
100 kD
75 kD
50 kD
37 kD
25 kD
20 kD
15 kD
Anti-Nopp140

Figure 9

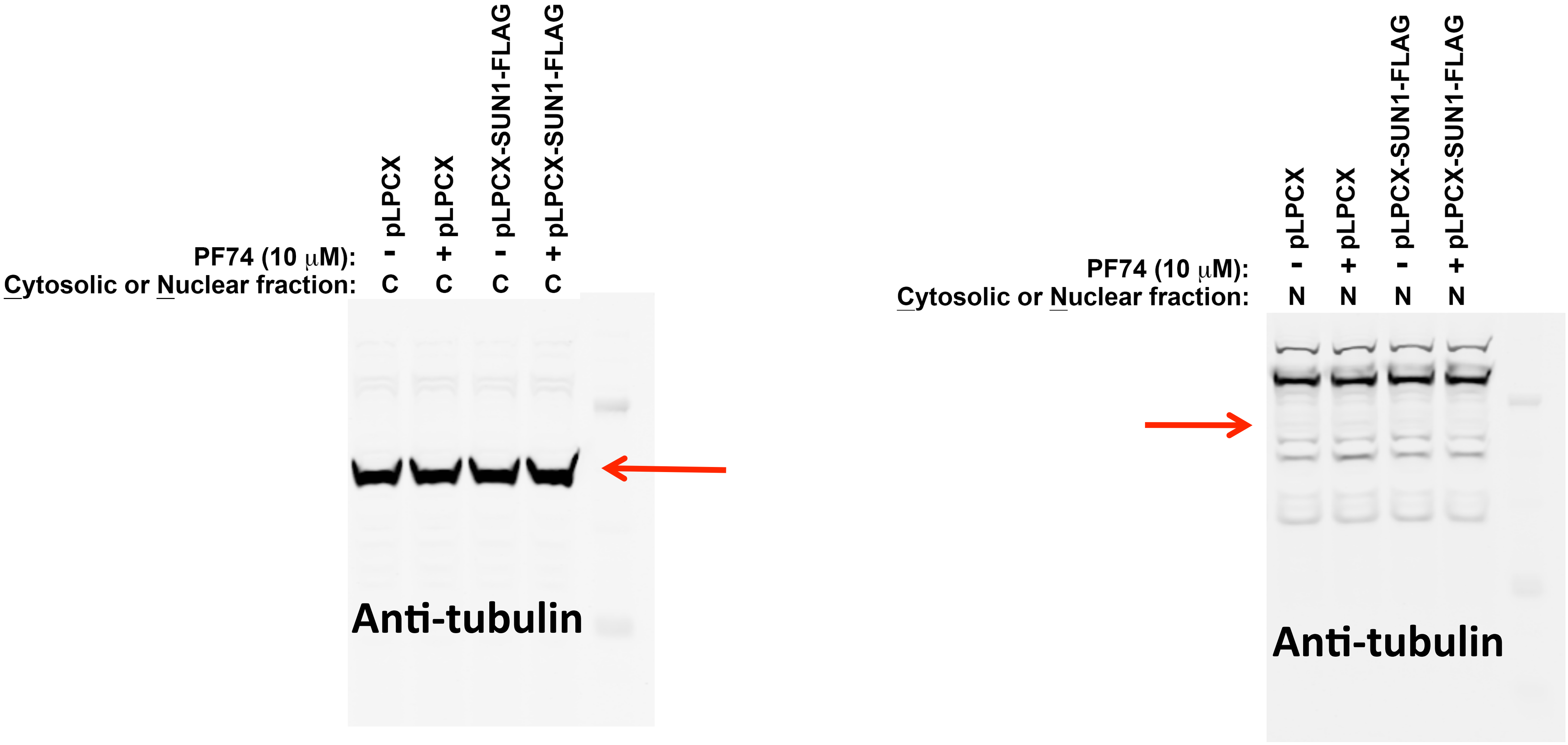

pLPCX
pLPCX
pLPCX-SUN1-FLAG
pLPCX-SUN1-FLAG
PF74 (10 μM): - + - +
Cytosolic or Nuclear fraction: C C C C
Anti-tubulin
pLPCX
pLPCX
pLPCX-SUN1-FLAG
pLPCX-SUN1-FLAG
PF74 (10 μM): - + - +
Cytosolic or Nuclear fraction: N N N N
Anti-tubulin

Figure 10B

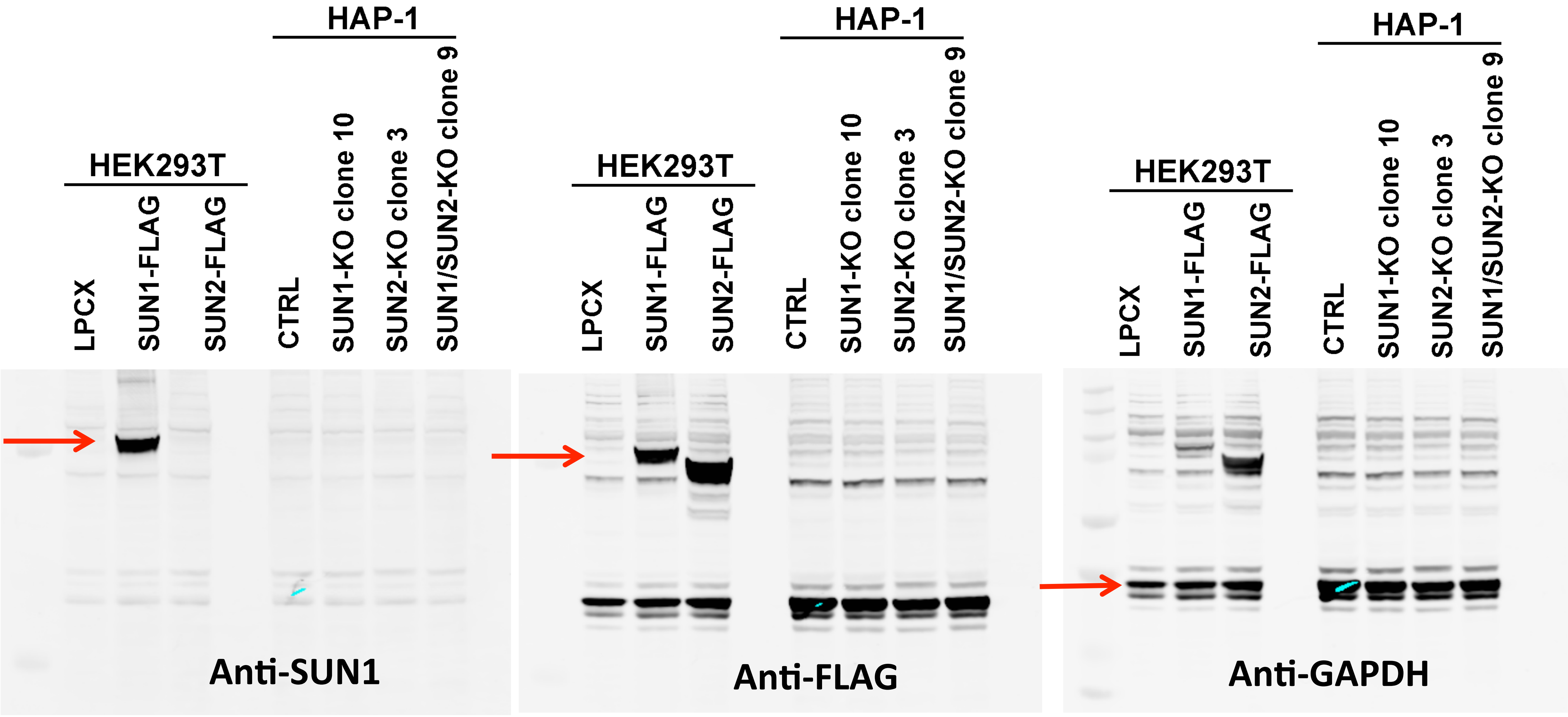
HAP-1
HEK293T
LPCX
SUN1-FLAG
SUN2-FLAG
CTRL
SUN1-KO clone 10
SUN2-KO clone 3
SUN1/SUN2-KO clone 9
Anti-SUN1
Anti-FLAG
Anti-GAPDH

Figure 10B

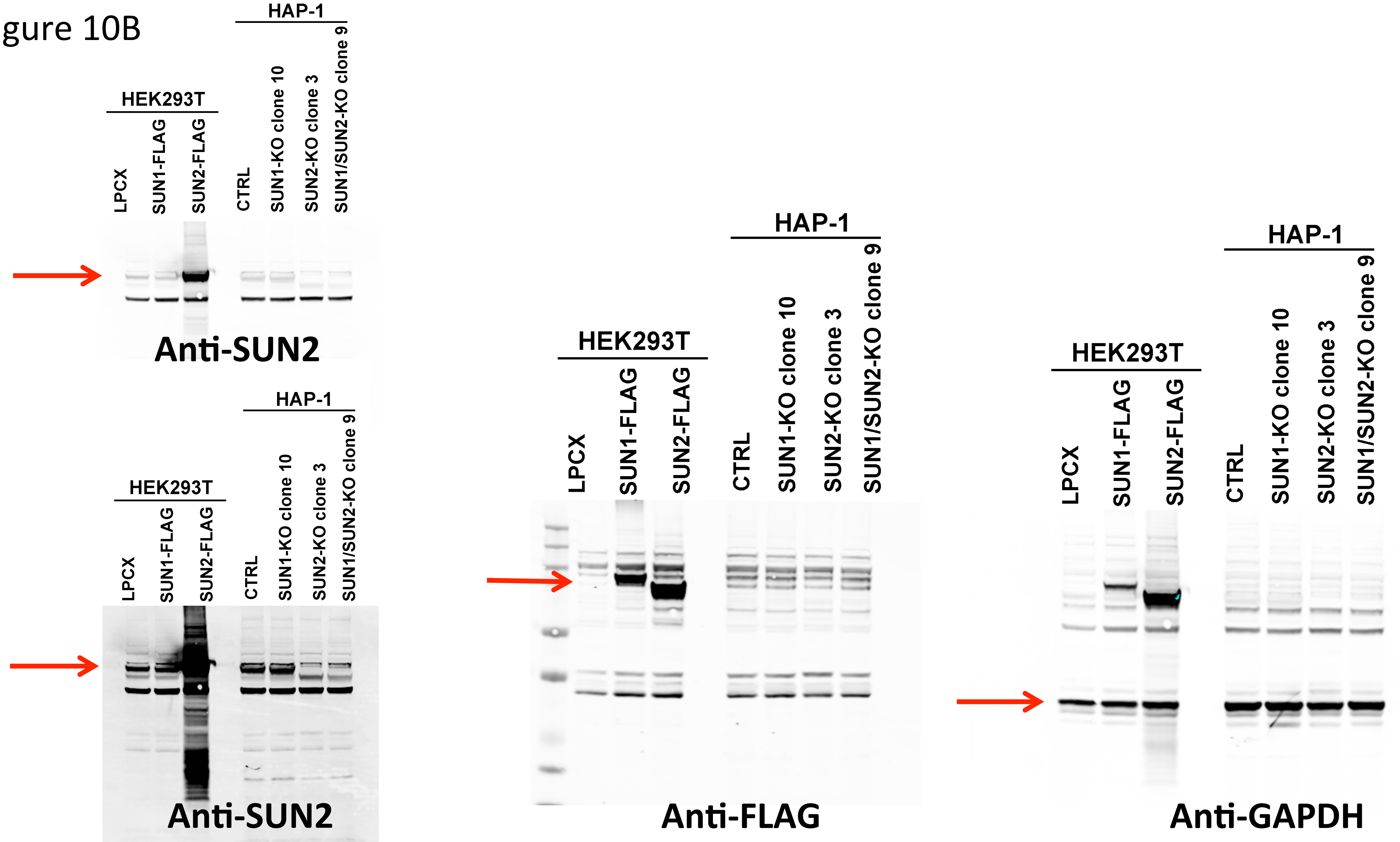

HAP-1
HEK293T
LPCX
SUN1-FLAG
SUN2-FLAG
CTRL
SUN1-KO clone 10
SUN2-KO clone 3
SUN1/SUN2-KO clone 9
Anti-SUN2
HAP-1
HEK293T
LPCX
SUN1-FLAG
SUN2-FLAG
CTRL
SUN1-KO clone 10
SUN2-KO clone 3
SUN1/SUN2-KO clone 9
Anti-SUN2
HAP-1
HEK293T
LPCX
SUN1-FLAG
SUN2-FLAG
CTRL
SUN1-KO clone 10
SUN2-KO clone 3
SUN1/SUN2-KO clone 9
Anti-FLAG
HAP-1
HEK293T
LPCX
SUN1-FLAG
SUN2-FLAG
CTRL
SUN1-KO clone 10
SUN2-KO clone 3
SUN1/SUN2-KO clone 9
Anti-GAPDH
